# Genomic Identification and Biochemical Characterization of Methyl Jasmonate (MJ)-Inducible Terpene Synthase Genes in Lettuce (*Lactuca sativa* L. cv. Salinas)

**DOI:** 10.3390/plants15010055

**Published:** 2025-12-24

**Authors:** Akhileshwar Singh, Moon-Soo Chung, Seung Sik Lee, Byung Yeoup Chung, Sungbeom Lee

**Affiliations:** 1 Advanced Radiation Technology Institute (ARTI), Korea Atomic Energy Research Institute (KAERI), Jeongeup 56212, Republic of Korea; akhil26@kaeri.re.kr (A.S.); mschung@kaeri.re.kr (M.-S.C.); sslee@kaeri.re.kr (S.S.L.); bychung@kaeri.re.kr (B.Y.C.); 2 Department of Radiation Science, University of Science and Technology (UST), Daejeon 34113, Republic of Korea

**Keywords:** *Lactuca sativa*, lettuce, terpene synthase, methyl jasmonate, monoterpenes, sesquiterpenes, headspace volatile

## Abstract

Terpenes are diverse plant metabolites with essential ecological and physiological functions, yet their biosynthetic regulation in lettuce (*Lactuca sativa* L.) remains poorly understood. By integrating volatile profiling, genome-wide identification, and biochemical characterization of terpene synthase (TPS) genes, we elucidated how methyl jasmonate (MJ) induces terpene formation in lettuce seedlings. Headspace analysis of 10-day-old seedlings revealed that while mock-treated tissues emitted no detectable volatiles, MJ elicitation triggered the de novo production of a terpene blend dominated by (*E*)-β-ocimene (9.3–14.6%), (*E*)-β-caryophyllene (37.2–46.9%), and caryophyllene oxide (26.2–41.4%). A genome-wide search identified 54 putative LsTPS genes, often clustered with prenyl transferases or cytochrome P450 genes. Gene expression assays revealed 17 MJ-responsive *LsTPS* genes; among them, *LsTPS21*, *LsTPS23*, *LsTPS28*, *LsTPS51*, and *LsTPS52* showed strong (>200-fold) induction, with *LsTPS52* exceeding a 20,000-fold increase. Functional characterization of six recombinant enzymes demonstrated diverse substrate specificities: LsTPS8 as an α-copaene synthase, LsTPS16 as a linalool synthase, LsTPS24 as an (*E*)-nerolidol synthase, LsTPS21 and LsTPS23 as (*E*)-β-ocimene synthases, and LsTPS10 as an (*E*)-β-caryophyllene synthase. Phylogenetic analyses confirmed conserved domains characteristic of the TPS-a and TPS-b subfamilies. This study presents the first comprehensive framework for MJ-induced terpene biosynthesis in lettuce, offering new insights into Asteraceae terpenoid metabolism.

## 1. Introduction

Terpenes are diverse plant metabolites that regulate crucial physiological processes and ecological interactions, functioning as antimicrobial agents or signaling molecules that activate defense responses and attract natural enemies of herbivores [1,2,3]. These compounds are typically localized in specialized structures such as glandular trichomes and laticifers [4,5], allowing for their release as volatile organic compounds that mediate plant communication, including pollinator attraction and stress defense [6,7,8,9]. Classified by their number of isoprene units, they range from hemiterpenes (C_5_), monoterpenes (C_10_), and sesquiterpene (C_15_), and diterpene (C_20_) to large polymers and atypical homoterpenes [10], with their biosynthesis driven by the terpene synthase (TPS) gene family, which is phylogenetically divided into functional subfamilies, like TPS-a for sesquiterpenes and TPS-b for monoterpenes [11,12]. The Asteraceae family is well-known for producing bioactive terpenoids [13,14,15,16,17,18,19,20], and while specific lettuce genes such as germacrene A synthase (GAS1 and GAS2) and diterpene synthases have been functionally characterized, a significant portion of the TPS repertoire remains unexplored [21,22]. Detailed biosynthetic studies on sesquiterpene lactones in lettuce [23] and chicory have mapped the pathway from germacrene A formation to (+)-costunolide [17,24,25]. Recent studies employing CRISPR/Cas9 gene editing have confirmed the involvement of several P450 enzymes that function as a kauniolide synthase and a lactucin synthase, which regiospecifically hydroxylates 8-deoxylactucin to generate lactucin in chicory confirming the role of cytochrome P450 enzymes in generating downstream guaianolides like lactucin [26,27].

Terpene biosynthesis regulation in response to stress is closely linked to jasmonate signaling. Specifically, methyl jasmonate (MJ)—a chemical proxy of herbivore—acts as a primary elicitor of TPS gene expression and volatile emission [28]. MJ treatment triggers the de novo synthesis and release of monoterpenes and sesquiterpenes, which serve as both direct defenses and indirect signals recruiting natural enemies of herbivores or inhibiting pathogens [29,30,31,32,33]. Recent genomic resources for lettuce now enable the systematic identification and functional classification of TPS gene families [34], yet comprehensive MJ-integrated analyses of gene expression, volatile emissions, and enzyme function remain largely unexplored in lettuce, a major leafy vegetable belonging to the Asteraceae.

In this study, headspace volatile analysis combined with genomic and transcriptomic, and biochemical approaches was employed to elucidate MJ-induced terpene biosynthesis in lettuce seedlings. We first profiled volatile emissions from lettuce shoots following MJ elicitation, revealing the de novo synthesis of a terpene blend dominated by (*E*)-β-ocimene, (*E*)-β-caryophyllene, and caryophyllene oxide. Subsequently, 54 putative TPS genes were identified from the lettuce genome, among which a subset exhibited strong MJ-responsiveness based on RT-PCR and qRT-PCR analyses. Several highly induced *LsTPSs* were cloned, heterologously expressed, and functionally characterized to determine their enzymatic activities and substrate specificities. Phylogenetic analysis and predicted subcellular localization further predicted evolutionary relationships and potential compartmentalization of TPS function. We successfully linked volatile profiling with genomic data to identify *LsTPS21*, *LsTPS23*, and *LsTPS10* as the key metabolic players that shape the volatile landscape of lettuce in response to abiotic stress mimicking herbivory.

## 2. Results

### 2.1. Methyl Jasmonate (MJ)-Induced Volatile Terpenes in L. sativa Seedlings

**Figure 1:**
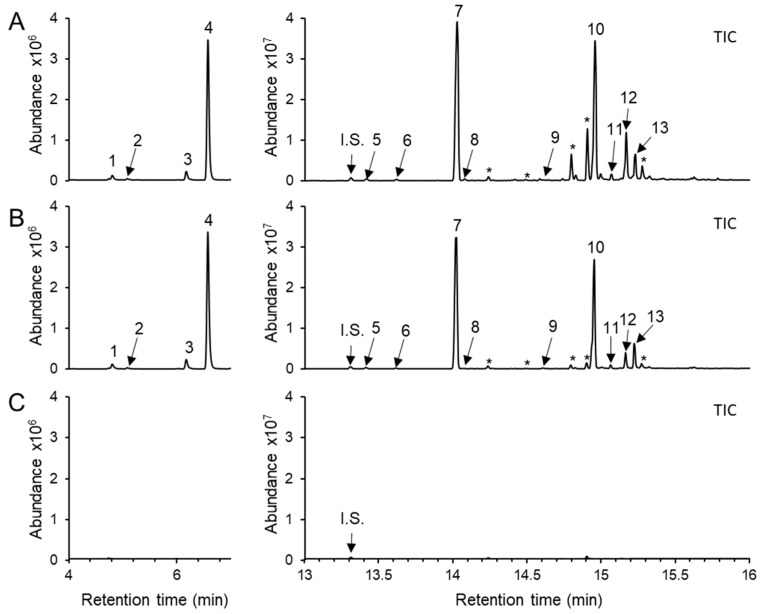
Headspace volatile terpenoids emitted from methyl jasmonate (MJ)-treated lettuce *(Lactuca sativa* cv. Salinas) seedlings. Only aerial part (approx. 1 g FW) was harvested from 10-day-old lettuce seedlings and enclosed in a glass vial (20 mL) at 12 h after MJ treatment with (**A**) 1 mM, (**B**) 0.5 mM, and (**C**) Mock (2% ethanol in water). The volatiles were analyzed by solid-phase microextraction-gas chromatography-mass spectrometry (SPME-GC-MS). Each peak was identified using authentic standards, Wiley Registry (12th edition) and the 2020 NIST library. 1, sabinene; 2, β-myrcene; 3, D-limonene; 4, β-ocimene; 5, guaia-4,11-diene; 6, copaene; 7, (*E*)-β-caryophyllene; 8, 10,10-dimethyl-2,6-dimethylenebicyclo[7.2.0] undecane; 9, (*E*)-α-farnesene; 10, caryophyllene oxide; 11, humulene epoxide; 12, 11,11-dimethyl-4,8-dimethylenebicyclo[7.2.0]undecan-3-ol; 13, hydroxycaryophyllene; and *, unknown terpenoid. Three biologically independent samples were analyzed and a representative chromatogram with similar results is shown. I.S.—Internal standard (1-bromodecane).

**Figure 2:**
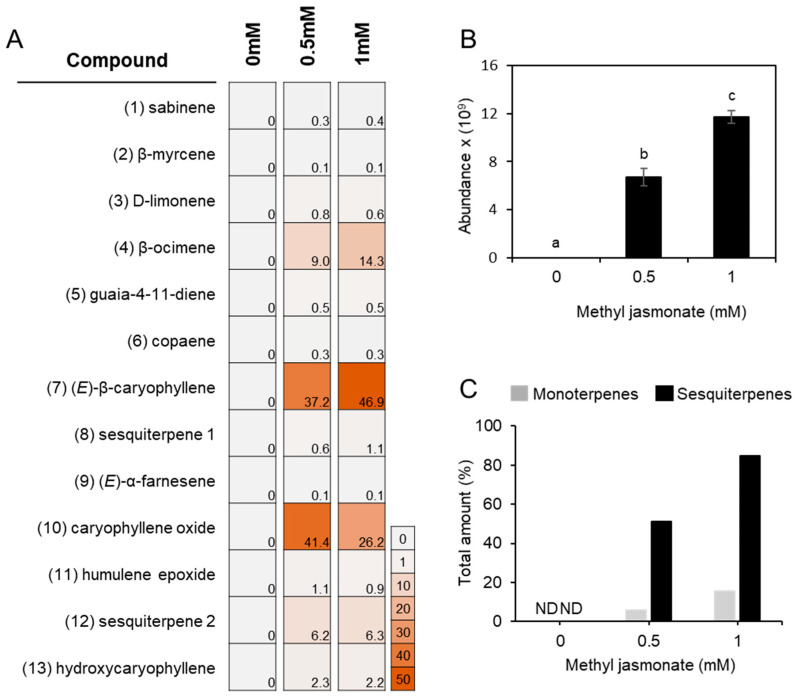
Relative abundance of volatile terpenoids released from MJ-treated lettuce seedlings. (**A**) Percentage distribution of the compounds detected from the shoot (approx. 1 g FW) of lettuce seedlings in response to methyl jasmonate (MJ) treatment. Each peak area of the terpenoids shown in Figure 1 was combined and employed to estimate the relative amount of a compound in percentile. A number preceding a compound name corresponds to a peak number shown in Figure 1. Sesquiterpene 1: 10,10-dimethyl-2,6-dimethylenebicyclo[7.2.0]undecane; Sesquiterpene 2: 11,11,11-dimethyl-4,8-dimethylenebicyclo[7.2.0]undecan-3-ol. (**B**) MJ-dose dependent production of total terpenoids collected from the shoots. The abundance of all terpene compounds at each treatment was combined. Data represent the mean ± SE from three biologically independent replicates. Statistical significance was determined by one-way ANOVA with Tukey’s HSD post hoc test. Different letters indicate significant differences at *p* < 0.05. (**C**) MJ-dose dependent production of mono- and sesquiterpenes. The amounts of terpenoids at 0.5 mM treatment was calculated as a percentage relative to the total abundance detected at 1 mM (shown in Figure 2B), which was normalized to 100%. ND, not detected.

To determine the volatile terpene profile in *L. sativa* seedlings, headspace volatiles were collected from the aerial part of 10-day-old seedlings and analyzed using a solid phase microextraction–gas chromatography–mass spectrometry (SPME–GC–MS). No volatile terpene compounds were detected in untreated shoot tissues under our analytical condition (Figure 1C). To induce terpene biosynthesis, MJ was applied as an elicitor, as MJ is well known to enhance terpene synthase (TPS) gene expression and terpenoid accumulation in several plant species, including members of the Asteraceae [29,30,35,36,37]. Ten-day-old lettuce seedlings were treated with 0.5 mM or 1 mM MJ for 12 h, and a blend of volatile terpenes was de novo detected in the shoots at both concentrations with no discernible phenotypic differences (Figure 1A,B and Figure S12). Upon 0.5 mM MJ treatment, four monoterpenes such as sabinene (0.3 ± 0.1%), β-myrcene (0.1 ± 0.0%), D-limonene (0.8 ± 0.1%), and β-ocimene (9.3 ± 1.7%) were detected. Increasing the MJ concentration to 1 mM did not substantially alter the relative abundance of these monoterpenes, except for β-ocimene, which released from 9.3 ± 1.7% to 14.6 ± 5.9% (Figure 2A). For sesquiterpenes, (*E*)-β-caryophyllene was the most strongly induced compound among the 9 sesquiterpenoids detected, followed by caryophyllene oxide. The relative proportions of the 9 sesquiterpenoids at 0.5 mM and 1 mM MJ, respectively, were as follows: copaene (0.3 ± 0.0% and 0.3 ± 0.1%), (*E*)-β-caryophyllene (37.2 ± 7.8% and 46.9 ± 3.1%), 10,10-dimethyl-2,6-dimethylenebicyclo[7.2.0]undecane (0.6 ± 0.5% and 1.1 ± 1.4%), (*E*)-α-farnesene (0.1 ± 0.1% and 0.1 ± 0.0%), guaia-4-11-diene (0.5 ± 0.1% and 0.5 ± 0.0%), caryophyllene oxide (41.4 ± 7.9% and 26.2 ± 5.5%), humulene epoxide (1.1 ± 0.2% and 0.9 ± 0.4%), 11,11-dimethyl-4,8-dimethylenebicyclo[7.2.0]undecan-3-ol (6.2 ± 2.6% and 6.3 ± 3.9%), and hydroxycaryophyllene (2.3 ± 2.6% and 2.2 ± 3.9%). Notably, oxidation form of caryophyllene was more abundant than (*E*)-β-caryophyllene at 0.5 mM MJ treatment, but decreased relative to (*E*)-β-caryophyllene at 1 mM MJ (Figure 2A). The total amount of emitted terpenoids increased in an MJ-concentration-dependent manner, with sesquiterpenes showing a greater fold change than monoterpenes (Figure 2B,C).

### 2.2. Genomic Identification of Putative L. sativa TPS Genes

**Figure 3:**
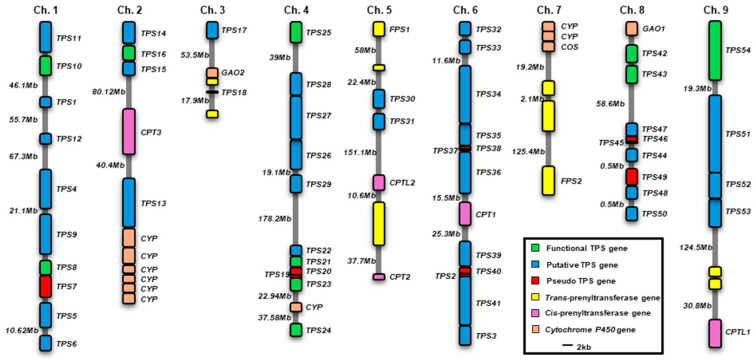
Chromosomal distribution of lettuce terpene synthase genes (*TPSs*). The organization of *TPSs* and other related genes on the lettuce chromosomes. CPT, *cis*-prenyl transferase; CPTL, *cis*-prenyl transferase like; CPS, *ent*-copalyl diphosphate synthase; FPS, Farnesyl diphosphate synthase; GAO, germacrene A oxidase; COS, costunolide synthase; CYP, cytochrome P450. Functional *TPSs* include both functionally characterized genes in this work and previously reported in the studies.

A total of 54 putative terpene synthase genes were retrieved from the Phytozome database. Two previously characterized germacrene A synthase genes (*LsGAS1* and *LsGAS2*) were assigned as *LsTPS43* and *LsTPS42*, respectively [21]. In addition, two diterpene cyclases, *ent*-copalyl diphosphate synthase 1 (*LsCPS1*) and *ent*-kaurene synthase (*LsKS*), were designated as *LsTPS54* and *LsTPS25*, respectively (Table S1) [22]. A total of 54 LsTPS genes were unevenly distributed across nine chromosomes, with no TPS genes detected on chromosome 7 (Figure 3). Chromosome 6 harbored the largest cluster, containing *LsTPS2*, *LsTPS3*, and *LsTPS32*–*LsTPS41*, whereas several chromosomes (e.g., Chr. 3, Chr. 5, and Chr. 9) contained only a limited number of LsTPS genes. Functional LsTPS genes (green) and putative *LsTPS* genes (blue) frequently occurred in tandem arrays, suggesting lineage-specific local gene duplication. Eleven pseudo-TPS genes (red) showed either the atypical short coding sequences or the absence of a start codon (Table S1) and were localized only in a few small clusters on chromosomes 1, 3, 4, 6, and 8 (Figure 3). Notably, genes involved in terpene precursor biosynthesis, such as *cis*-prenyl transferase (CPT) and farnesyl pyrophosphate synthase (FPS), were frequently colocalized with *LsTPS* loci. Specific examples include *CPT3* on Chr. 2, *CPT1* on Chr. 6, and *CPTL1* on Chr. 9. Likewise, several P450 genes (orange) were also located adjacent to *LsTPS* loci on chromosomes 2, 7, and 8. This genomic co-localization of *LsTPSs*, precursor-synthesizing enzymes, and putative downstream modifying enzymes suggests a potential spatial organization of terpene biosynthetic components in lettuce (Figure 3).

### 2.3. MJ-Inducible Expression of LsTPS Genes Analyzed by a Semi-Quantitative and Quantitative RT–PCR

Lettuce shoot emits a bouquet of headspace volatile terpenoids in response to MJ treatment (Figure 1). To identify MJ-responsive *LsTPS* genes, we examined steady-state transcript level of 43 putative *LsTPS* genes excluding 11 pseudogenes, which were predicted from the Phytozome database using semi-quantitative RT–PCR. Seventeen genes—*LsTPS4*, *LsTPS5*, *LsTPS8*, *LsTPS10*, *LsTPS15*, *LsTPS16*, *LsTPS21*, *LsTPS23*, *LsTPS24*, *LsTPS26*, *LsTPS28*, *LsTPS34*, *LsTPS42*, *LsTPS43*, *LsTPS50*, *LsTPS51*, and *LsTPS52*—showed inducible transcript accumulation in response to MJ treatment (Figure S1). Among these, nine genes (*LsTPS15*, *LsTPS16*, *LsTPS21*, *LsTPS23*, *LsTPS24*, *LsTPS28*, *LsTPS50*, *LsTPS51*, and *LsTPS52*) exhibited particularly strong induction (Figure S1).

**Figure 4:**
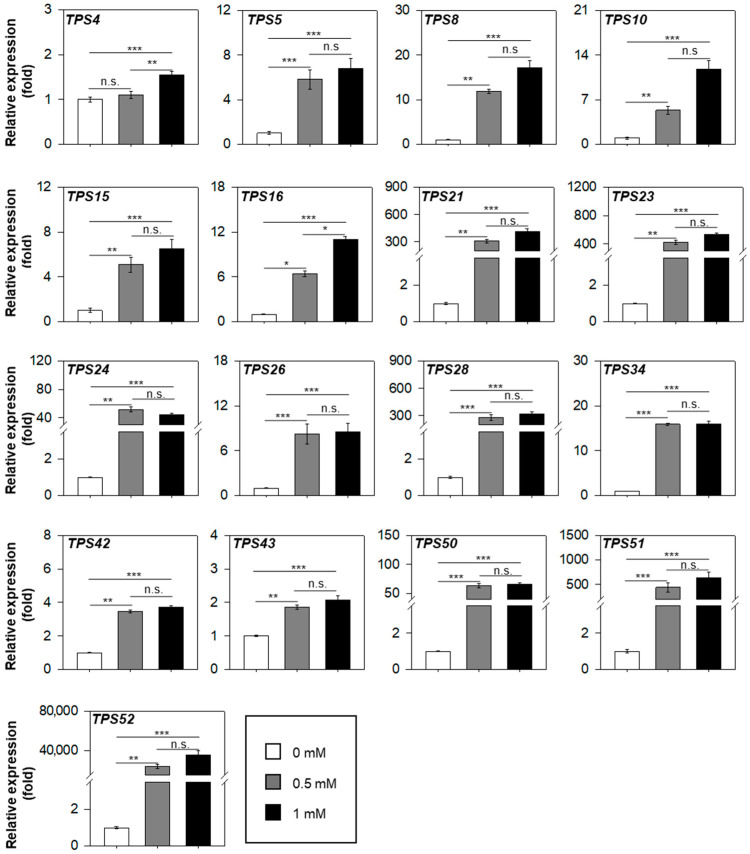
Quantitative reverse transcription (qRT)–polymerase chain reaction (PCR) assay of MJ-inducible *LsTPS* genes. A *LsTub* was used as an endogenous reference gene. Shoot tissue of 10-day-old lettuce seedlings were foliar sprayed until saturation with methyl jasmonate at concentrations of Mock (2% ethanol in water), 0.5 mM, and 1 mM for 12 h. The expression levels were normalized to the mock treatment, which was set to 1 (fold). Data represent the mean ± SE (*n* = 3) in triplicate. Statistical significance was determined by the non-parametric Kruskal–Wallis test followed by Dunn’s post hoc test (n.s.: not significant; * *p* < 0.05; ** *p* < 0.01; *** *p* < 0.001).

To quantify the transcript level of these MJ-responsive TPS genes, we performed quantitative RT–PCR assay, normalizing expression values to the mock treatment (set to 1-fold) (Figure 4). Modest induction (<20-fold) was observed for *LsTPS4*, *LsTPS5*, *LsTPS8*, *LsTPS10*, *LsTPS15*, *LsTPS16*, *LsTPS26*, *LsTPS34*, *LsTPS42*, and *LsTPS43* with *LsTPS4*, *LsTPS42*, and *LsTPS43* showing less than 5-fold changes. In contrast, *LsTPS21*, *LsTPS23*, *LsTPS28*, *LsTPS51*, and *LsTPS52* displayed dramatic induction (>200-fold). Remarkably, *LsTPS52* increased by more than 20,000-fold after MJ treatment. Moreover, seven TPS genes (*LsTPS8*, *LsTPS10*, *LsTPS16*, *LsTPS21*, *LsTPS23*, *LsTPS42*, and *LsTPS52*) showed an MJ-dose dependent expression pattern.

### 2.4. Functional Characterization of MJ-Inducible LsTPS

All seventeen MJ-inducible *LsTPS* genes were initially selected for cloning and in vitro functional characterization using five prenyl diphosphate substrates: geranyl pyrophosphate (GPP) and neryl pyrophosphate (NPP) for monoterpene synthase assays; *E*,*E*-farnesyl pyrophosphate (FPP) and *Z*,*Z*-FPP for sesquiterpene synthase assays; and geranylgeranyl pyrophosphate (GGPP) for diterpene synthase assays. Full-length cDNAs of six *LsTPSs* (*LsTPS8*, *LsTPS10*, *LsTPS16*, *LsTPS21*, *LsTPS23*, and *LsTPS24*) were successfully amplified by PCR, cloned, heterologously expressed in *E. coli*, and purified as soluble recombinant proteins. LsTPS42 and LsTPS43 were disregarded for functional characterization because those were previously reported as a germacrene A synthase gene [21]. The remaining nine LsTPSs could not be biochemically characterized due to limited cDNA availability or difficulties in obtaining soluble protein. We cloned and bidirectionally sequenced five *LsTPS* genes (i.e., *LsTPS8*, *LsTPS16*, *LsTPS21*, *LsTPS23*, and *LsTPS24*). Upon comparison with the Phytozome database, we identified sequence discrepancies in all five genes (Figure S2). These mismatches resulted in several amino acids substitutions in LsTPS8, LsTPS21, and LsTPS24 (Figures S3–S5), whereas no amino acid changes were observed in LsTPS10, LsTPS16 or LsTPS23. Among the six recombinant enzymes, catalytic activities were tested with four precursors (GPP, NPP, *E*,*E*-FPP and *Z*,*Z*-FPP). None of the enzymes showed detectable activity with GGPP.

**Figure 5:**
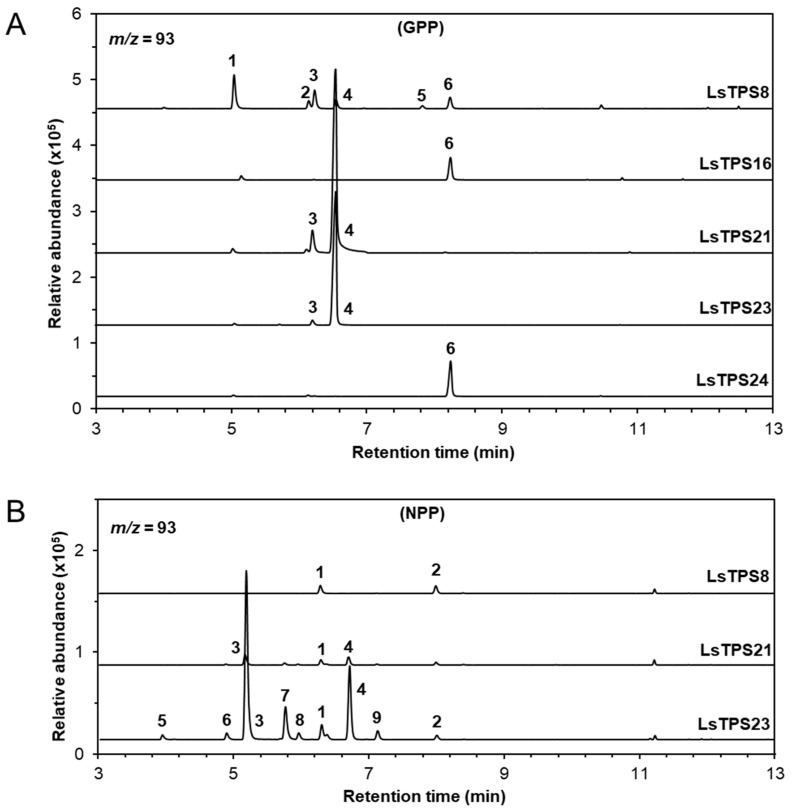
GC–MS analysis of monoterpenes catalyzed by MJ-inducible TPSs. Reaction products were identified by comparing their mass spectra and retention times with those of authentic standards, the Wiley Registry (12th edition), and the 2020 NIST Mass Spectral Library. Each TPS enzyme was reacted with (**A**) GPP as a substrate: 1, β-myrcene; 2, D-limonene; 3. (*Z*)-β-ocimene; 4, (*E*)-β-ocimene; 5, α-terpinolene; 6, linalool; or (**B**) NPP as a substrate: 1, D-limonene; 2, α-terpinolene; 3, β-myrcene; 4, (*E*)-β-ocimene; 5, β-thujene; 6, pseudolimonene; 7, 3-carene; 8, α-terpinene; and 9, γ-terpinene. Extracted ion chromatograms (*m*/*z* = 93) are shown. Identities, mass spectra and percentage distribution of each peak can be found in Figures S7 and S8 and Table S2.

**Figure 6:**
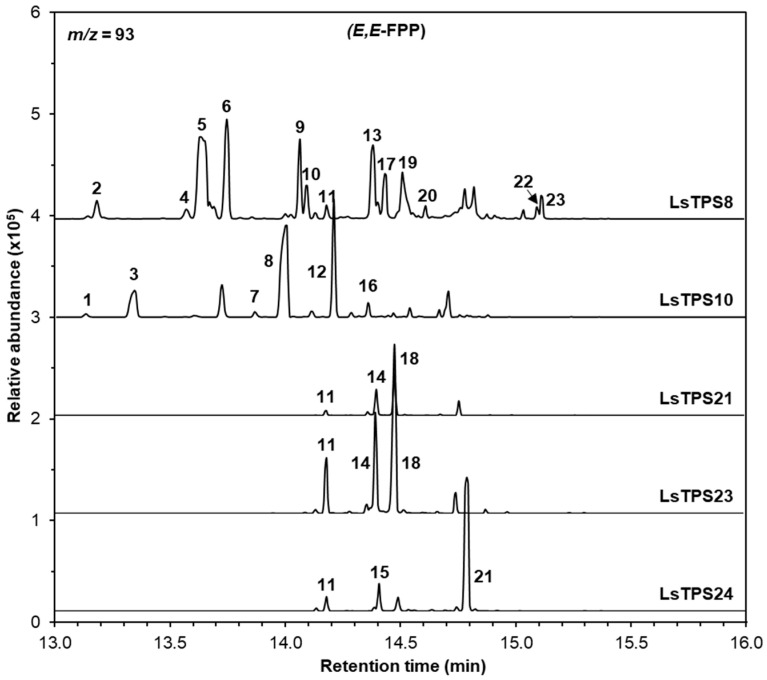
GC–MS analysis of sesquiterpenes catalyzed by MJ-inducible TPSs using *E*,*E*-FPP as a substrate. The reaction products were identified by comparing their mass spectra and retention times with those of authentic standards, the Wiley Registry (12th edition), and the 2020 NIST Mass Spectral Library: 1, silphiperfol-5-ene; 2, δ-eIemene; 3, 7-epi-silphiperfol-5-ene; 4, (+)-cyclosativene; 5, α-copaene; 6, β-elemene; 7, (*Z*)-β-caryophyllene; 8, (*E*)-β-caryophyllene; 9, γ-elemene; 10, (*Z*)-β-copaene; 11, (*E*)-β-farnesene; 12, α-humulene; 13, γ-muurolene; 14, (*E*)-α-bergamotene; 15, (*Z*,*E*)-α-farnesene; 16, aromandendrene; 17, (-)-germacrene D; 18, (*E*)-α-farnesene; 19, (-)-α-muurolene; 20, (-)-β-cadinene 21, (*E*)-nerolidol; 22, cubebol; and 23, (+)-viridiflorol. Extracted ion chromatograms (*m*/*z* = 93) are shown. Identities, mass spectra and percentage distribution of each peak can be found in Figure S9 and Table S2.

**Figure 7:**
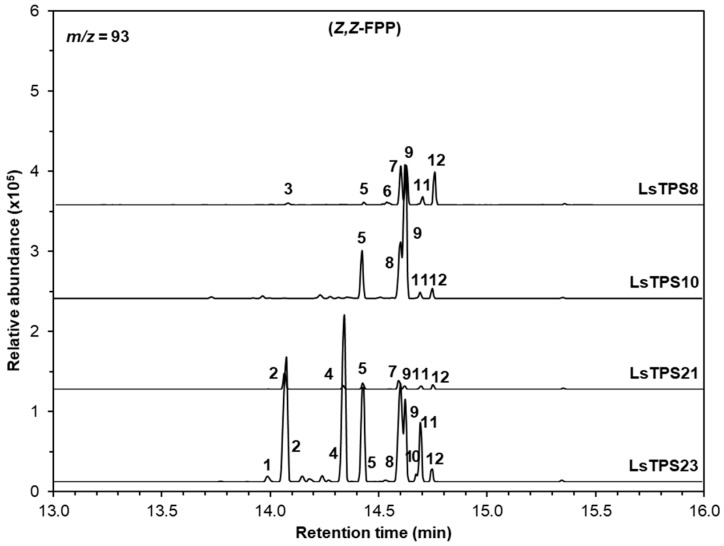
GC–MS analysis of sesquiterpenes catalyzed by MJ-inducible TPSs using *Z*,*Z*-FPP as a substrate. The reaction products were identified by comparing their mass spectra and retention times with those of authentic standards, the Wiley Registry (12th edition), and the 2020 NIST Mass Spectral Library: 1, (*E*)-β-caryophyllene; 2, (*Z*)-β-farnesene; 3, (-)-α-cedrene; 4, (*Z*)-α-bergamotene; 5, γ-curcumene; 6, (-)-zingiberene; 7, (-)-β-bisabolene; 8, β-curcumene; 9, (*Z*)-γ-bisabolene; 10, β-sesquiphellandrene; 11, (*E*)-γ-bisabolene; and 12, (*E*)-α-bisabolene. Extracted ion chromatograms (*m*/*z* = 93) are shown. Identities, mass spectra and percentage distribution of each peak can be found in Figure S10 and Table S2.

Recombinant LsTPS8 exhibited monoterpene synthase activity with GPP, producing β-myrcene (1) as the major product along with D-limonene (2), (*Z*)-β-ocimene (3), (*E*)-β-ocimene (4), α-terpinolene (5), and linalool (6). When NPP was used as a substrate, product formation was restricted to D-limonene (2) and α-terpinolene (5) (Figure 5). With *E*,*E*-FPP as a substrate, LsTPS8 produced a wide array of thirteen sesquiterpenes, including β-elemene (6) (a Cope-rearrangement product of germacrene A; 15.6%) and α-copaene (5) (29.6%) as the major compounds (Figure 6), hereafter referred to as α-copaene synthase based on the absence of signal peptides. In contrast, when *Z*,*Z*-FPP was employed, multiple bisabolene species were produced, including (-)-β-bisabolene (7), (*Z*)-γ-bisabolene (9), (*E*)-γ-bisabolene (11), and (*E*)-α-bisabolene (12) along with minor amount of α-cedrene (3), γ-curcumene (5), zingiberene (6) (Figure 7). LsTPS16 has highly specific substrate preferences, generating linalool (6) from GPP, with no detectable reaction products with NPP (Figure 5), hereafter designated linalool synthase. LsTPS24 converted *E*,*E*-FPP predominantly into (*E*)-nerolidol (21) (82.3%), accompanied by small amounts of farnesenes (Figure 6 and Figure S9), hereafter referred to as (*E*)-nerolidol synthase as it lacks signal peptides. Both LsTPS21 and LsTPS23 accepted GPP, producing exclusively (*E*)-β-ocimene (4) with trace amount of (*Z*)-β-ocimene (3), hereafter designated (*E*)-β-ocimene synthase, reflecting the presence of signal peptides; however, their product profiles with NPP were notably distinct. LsTPS23 yielded β-myrcene (3) (51.5%) as the major product along with nine additional monoterpenes (Figure 5 and Figure S8). In contrast, LsTPS21 produced only trace amounts of D-limonene (1), β-myrcene (3), and (*E*)-β-ocimene (4) from NPP.

LsTPS21 catalyzed *E*,*E*-FPP to produce (*E*)-β-farnesene (11), (*E*)-α-bergamotene (14), and the major product (*E*)-α-farnesene (18) (55.7%), which were similarly observed when LsTPS23 was employed (Figure 6). When supplied with *Z*,*Z*-FPP, LsTPS23 generated (*Z*)-α-bergamotene (4) (25.7%) and (*Z*)-β-farnesene (2) (22.7%) as the predominant products along with several minor compounds (Figure 7 and Figure S10). However, when LsTPS21 was used, (*Z*)-β-farnesene (2) (40.6%) was the major product with negligible amount of γ-curcumene (5) and several bisabolene isomers. Unlike other sesquiterpene synthases analyzed in this study, LsTPS10 displayed exclusive activity with both isomers of FPP, producing (*E*)-β-caryophyllene (8) (50.8%) using *E*,*E*-FPP, and (*Z*)-γ-bisabolene (9) (51.3%) from *Z*,*Z*-FPP (Figure 6 and Figure 7), hereafter referred to as (*E*)-β-caryophyllene synthase. Table S2 lists the details of the reaction products for all enzymes characterized in this study.

### 2.5. Protein Sequence Alignment and Phylogenetic Analysis of MJ-Inducible LsTPSs

**Figure 8:**
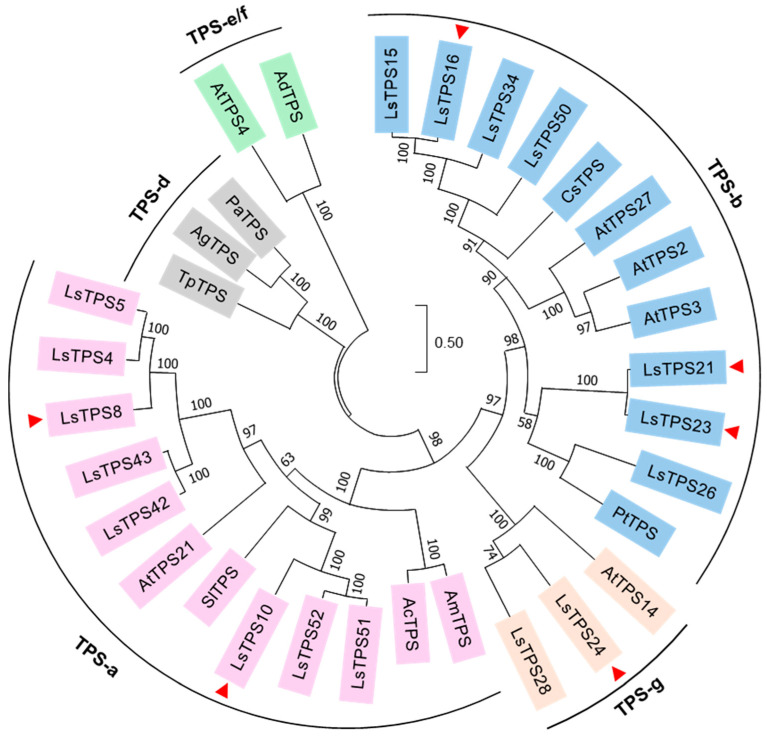
Phylogenetic analysis between lettuce TPSs and known TPSs in plants. A maximum-likelihood phylogenetic tree was generated using MEGA11 with a bootstrap value of 1000. Each TPS subgroup is shaded in different colors. Seventeen MJ-inducible TPSs in lettuce are considered to construct the tree. A red triangle represents the six TPSs biochemically characterized in this study. Abbreviations: Ls, *Lactuca sativa*; Pt, *Populus trichocarpa*; Pa, *Picea abies*; Ad, *Actinidia deliciosa*; Ac, *Aquilaria crassna*; Sl, *Solanum lycopersicum*; Am, *Aquilaria malaccensis*; Cs, *Cannabis sativa*; Ag, *Abies grandis*; Tp, *Thuja plicata*; and At, *Arabidopsis thaliana*.

The deduced amino acid sequences of the seventeen MJ-inducible LsTPS proteins were aligned with selected TPSs from Arabidopsis, tomato, poplar, Norway spruce, kiwi, agarwood, cannabis, grand fir, and redcedar using the CLUSTALW algorithm, and a phylogenetic tree was subsequently constructed. Eight LsTPS members were grouped into the TPS-a subfamily, which represents a highly divergent sesquiterpene synthase clade (Figure 8). Seven LsTPSs were assigned to the TPS-b clade, while two belonged to the TPS-g subfamily. No MJ-inducible LsTPS clustered with the TPS-d and the TPS-e/f subfamilies (Figure 8). The sequence alignment further revealed that the N-terminal R(R/S/P)(x)8W motif was conserved among LsTPS16, LsTPS47, LsTPS8, and LsTPS10, with a minor variation in LsTPS23, where isoleucine replaces the first arginine (Figure S6). The class I DDxxD motif and the C-terminal NSE/DTE [(N/D)DXX(S/T)XXXE] motif—both essential for divalent metal ion-dependent ionization of the prenyl diphosphate substrates—were also highly conserved across the LsTPS family (Figure S6).

## 3. Discussion

**Figure 9:**
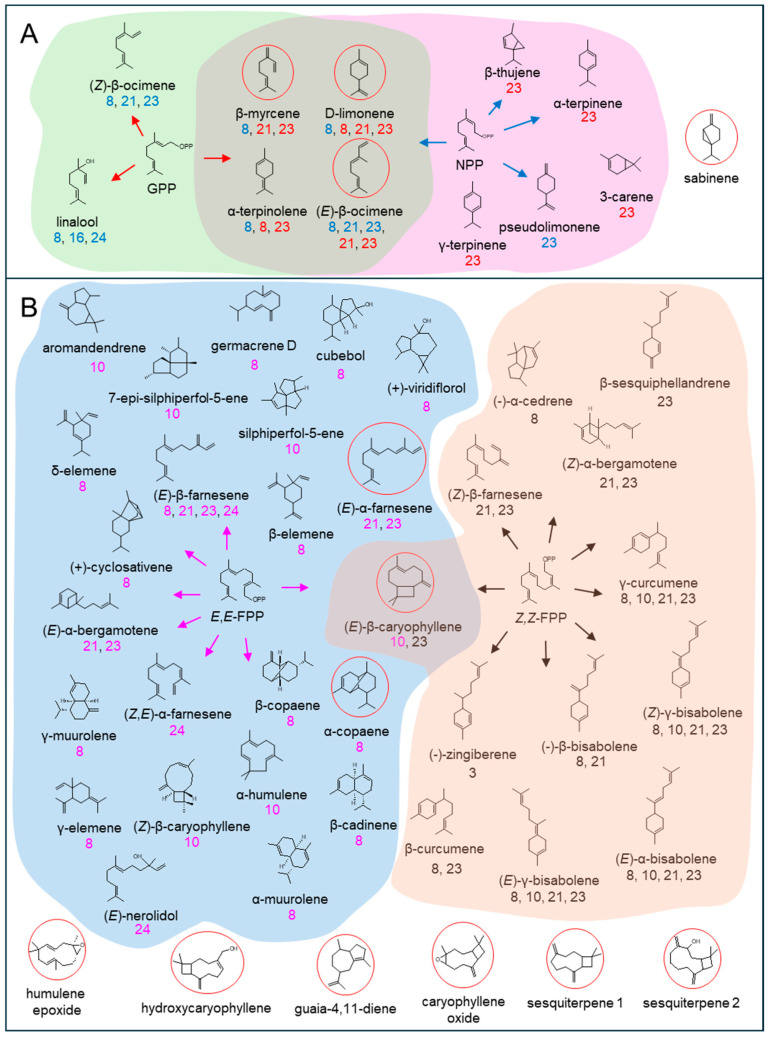
Terpene structures catalyzed by the six terpene synthases (TPSs) functionally characterized in this study. (**A**) GPP as a substrate: red arrows and TPS numbers, NPP as a substrate: blue arrows and TPS numbers; (**B**) *E*,*E*-FPP as a substrate: magenta arrows and TPS numbers; *Z*,*Z*-FPP as a substrate: black arrows and TPS numbers. β-elemene, γ-elemene, and δ-elemene are Cope-rearrangements of germacrene A, germacrene B, and germacrene C, respectively; circle in red, MJ-induced terpenes in lettuce shoot. The corresponding TPS were labeled beneath each compound in different colors.

Understanding how lettuce coordinates terpene metabolism under biotic stress requires an integrative approach. This necessitates combining volatile profiling with detailed functional analyses of the specific terpene synthase genes responsible for biosynthesis. Methyl jasmonate (MJ) treatment at 1 mM, which simulates herbivore attack, triggered the emission of a diverse terpene blend. This profile was dominated by (*E*)-β-ocimene (14.3%), (*E*)-β-caryophyllene (46.9%), and caryophyllene oxide (26.2%), whereas mock-treated plants emitted no detectable volatiles (Figure 1 and Figure S11). Previous studies reported induction of TPS genes at 0.1 mM MJ in lettuce; however, our inability to detect volatiles at this lower concentration prompted the use of higher MJ doses (>0.5 mM) to evoke a robust metabolic response [18]. The elicitation of (*E*)-β-ocimene emission aligns with its well-documented role as a volatile signaling molecule involved in indirect plant defense by attracting natural enemies of herbivores [32,38,39,40]. This biochemical response correlated strongly with the transcription activation of the (*E*)-β-ocimene synthase gene (*LsTPS23*) (>500-fold), highlighting a tight link between gene expression and volatile emission (Figure 4). Furthermore, our results indicate that lettuce emits a relatively narrow spectrum of monoterpenes upon MJ treatment. Remarkably, the catalytic activities of just two enzymes, LsTPS21 and LsTPS23, are sufficient to account for all detected monoterpenes, with the exception of sabinene (Figure 9 and Figure S11).

Among sesquiterpenes, (*E*)-β-caryophyllene was the predominant volatile induced by MJ in lettuce shoots (Figure 1). This compound is notable for its antimicrobial properties in other model systems, such as Arabidopsis floral tissues [41], and its signaling properties for indirect defense in insect-damaged maize root. It achieves these attributes by attracting entomopathogenic nematodes [42,43]. However, the comparatively modest fold induction (<14-fold) of *LsTPS10*, encoding (*E*)-β-caryophyllene synthase, suggests that final terpene accumulation is regulated beyond transcript abundance alone (Figure 4). In vitro functional assays revealed that LsTPS21 and LsTPS23 accepted all four tested prenyl pyrophosphate precursors, exhibiting considerable catalytic versatility. Their close genomic proximity and sequence identity (96.8% identity in nucleotide sequences) strongly indicates their origin through tandem duplication events during the course of gene evolution (Figure 3). In contrast, LsTPS10 displayed selective activity exclusively with two stereoisomers of FPPs, highlighting substrate specificity that may underpin biochemical differentiation among sesquiterpene synthases. Similarly, LsTPS16 showed a distinct substrate preference for GPP, implying the substrate-driven diversification of TPS enzyme function (Figure 5, Figure 6, Figure 7 and Figure 9). We found that the sesquiterpene synthases characterized in this study were responsible for the biosynthesis of three major terpene hydrocarbons—(*E*)-β-caryophyllene, (*E*)-α-farnesene, and α-copaene—which are released from MJ-elicited lettuce shoot (Figure 9). To further elucidate the molecular determinants of this enzyme plasticity, computational substrate-enzyme docking simulations offer a powerful tool. By correlating active site architecture with catalytic outcomes, these models can help explain the structural mechanisms that allow LsTPS21/23 to accept diverse substrates while restricting LsTPS10/16 to specific precursors.

Genomic analysis by De Bruyn et al. identified 47 putative TPS genes in the *L. sativa* ‘ZOBRA’ genome [18]. While our BLAST (https://phytozome-next.jgi.doe.gov/blast-search, accessed on 24 June 2025) search largely aligned with their findings, discrepancies were observed: five genes reported in their study were absent in our results, whereas *LsTPS1* (*Lsat_1_v5_gn_0_28161*), identified in our analysis, was missing from their list. These inconsistencies likely reflect cultivar-specific genomic variations or the use of divergent computational filtering criteria.

Furthermore, our qRT-PCR assays demonstrated that higher MJ concentrations (0.5–1 mM) induced a distinct subset of genes (*LsTPSs 21*, *23*, *24*, *28*, *50*, *51*, and *52*) compared to the lower concentration (0.1 mM) utilized by De Bruyn et al., which primarily upregulated *LsTPSs 6*, *8*, *24*, *51*, *52*, and *53* (Figure 4). We attribute these divergent expression profiles to differences in experimental design, specifically the choice of cultivar (Salinas vs. ZORBA), MJ dosage (>0.5 mM vs. 0.1 mM), and sampling time (12 h vs. 6 h).

Chromosomal mapping analysis further revealed typical clustering of *LsTPS* genes consistent with lineage-specific tandem duplications as a core evolutionary mechanism driving metabolic diversification. The co-localization of *LsTPS* genes with CPT, FPS, and P450 loci suggests a coordinated genomic organization. This arrangement links precursor biosynthesis, terpene formation, and downstream modifications, likely optimizing metabolic flux through the pathway (Figure 3). Although physical proximity alone does not confirm functional linkage, this organization is coherent with the biosynthetic structuring found in other specialized metabolite pathways, facilitating coordinated regulation and efficient terpenoid production. The data reveal that lettuce possesses a sophisticated terpene biosynthetic network that is structurally and functionally integrated.

Despite the insights gained from this study, several limitations must be acknowledged. First, volatile emission analyses were conducted under controlled MJ treatment conditions, which may not fully capture the complexity of plant responses in natural biotic stress environments. Second, while in vitro enzyme assays provided valuable insights into substrate specificity, in planta functional validation using gene knockout or overexpression is essential to confirm physiological roles. This is particularly relevant for LsTPSs 50, 51, and 52, which were prioritized due to their extraordinarily high induction upon MJ treatment. Unfortunately, these proteins proved insoluble in our bacterial expression system, preventing biochemical characterization. However, sequence homology analysis offers clues to their potential functions: LsTPS50 and LsTPS51 share high identity (76%) with β-caryophyllene synthases from *Artemisia annua* (accession no. Q8SA63) and *Tanacetum parthenium* (accession no. F8UL81), suggesting they may contribute to the substantial β-caryophyllene emission observed in this study. Similarly, LsTPS50 closely resembles *R*-linalool synthases from *A. annua* (accession no. Q9SPN0 and Q9SPN1).

To address these gaps, future research should prioritize in planta functional validation using gene editing technologies such as CRISPR-Cas9, to establish causal links between specific TPS genes and terpene-mediated defense traits. Furthermore, extending volatile profiling to ecologically relevant biotic stress conditions across multiple lettuce cultivars will help elucidate the natural variation and adaptive significance of terpene biosynthesis. Finally, structural and computational modeling studies could further clarify the molecular determinants of substrate specificity, facilitating targeted metabolic engineering.

## 4. Materials and Methods

### 4.1. Plant Materials and Chemicals

Lettuce (*Lactuca sativa* cv. Salinas) seeds were purchased from Reimer seeds (www.reimerseeds.com). The seeds were surface-sterilized with 2% (*v*/*v*) sodium hypochlorite for 15 min and washed with sterile distilled water prior to germinate on 0.7% (*w*/*v*) phytoagar solid medium containing 1× Murashige and Skoog salts (MS) medium with vitamins and 2% sucrose grown in a magenta box. Germinating seeds (ten seeds per a box) were incubated in dark in a growth room for 3 days and then transferred to the light condition (16 h/8 h = light/dark, 150 μmol min^−2^s^−1^, 22 °C) to grow further for 10 days (hereafter referred to as 10-day-old seedlings). All chemicals used in vitro enzyme assay were purchased from Sigma-Aldrich (St. Louis, MO, USA), unless otherwise stated. Geranyl pyrophosphate (GPP), neryl pyrophosphate (NPP), *trans*- and *cis*-farnesyl pyrophosphate (FPP) and geranylgeranyl pyrophosphate (GGPP) were purchased from Echelon Biosciences (Salt Lake City, UT, USA). Authentic standards of (*R*)-(+)-limonene, linalool, α-terpinolene, α-humulene, (*E*)-caryophyllene, ocimene mixture, β-elemene, (+)-cyclosativene, nerolidol mix, and farnesene mixture were purchased from Sigma-Aldrich (St. Louis, MO, USA). α-Copaene and bisabolene mix were obtained from American Custom Chemicals Corporation (San Diego, CA, USA) and Alfa Aesar (Thermo Fisher Scientific, Waltham, MA, USA), respectively.

### 4.2. Genome-Wide Identification of Putative LsTPS Genes

Putative TPS genes in lettuce were first identified through BLASTP searches in the Phytozome (https://phytozome-next.jgi.doe.gov/info/Lsativa_V8 (accessed on 24 July 2023)) [44] using protein sequences of six experimentally characterized *Arabidopsis thaliana* TPS enzymes including monoterpene-, sesquiterpene-, and diterpene synthases (NP_001328553.1, NP_001329376.1, AEE33784.1, NP_001185286.1, NP_001190374.1, and AEE77075.1) as queries. The resulting candidates were further validated by BLAST analyses against the NCBI database. A total of 54 putative TPS genes were identified in the lettuce genome, and they were designated as *LsTPS1* to *LsTPS54* based on their order, assigned by Reyes-Chin-Wo et al. (Table S1) [34,45,46].

### 4.3. Methyl Jasmonate (MJ) Treatment

Ten-day-old lettuce seedlings grown in a magenta box were foliar sprayed with MJ (Sigma-Aldrich) at a concentration of 0.5 mM and 1 mM until leaves were saturated (approximately 10 sprays), and 2% ethanol was sprayed as a control treatment under a laminar flow. Only aerial parts of the seedlings were collected at 12 h after the MJ treatment. The collected tissues were subsequently placed in a solid-phase microextraction (SPME) glass vial for a volatile analysis or were stored at −80 °C until use.

### 4.4. Collection and Identification of MJ-Inducible Volatile Terpenoids

Headspace volatile compounds emitted from MJ-treated lettuce seedlings were analyzed according to Chung et al. with minor modifications [47]. Leaves of MJ-treated lettuce seedlings (approximately 1 g fresh weight of 10-day-old seedlings) were enclosed in a 20 mL glass vial containing a cotton swab at the bottom with 0.5 mL sterile distilled water and 20 ng of 1-bromodecane as an internal standard. The vials were incubated under constant light condition at 22 °C for 24 h in an in-house growth room and headspace volatiles were collected using a SPME fiber [100 µm polydimethylsiloxane (PDMS), fused silica 24 Ga) (Supelco Inc., Bellefonte, PA, USA)] for 1 h. The fiber was then inserted into the injection port of a gas chromatography-mass spectrometry (GC-MS) (Agilent GC system, 7890A; MSD, 5957C) equipped with a DM-5MS capillary column (Agilent Technologies, Santa Clara, CA, USA) of 0.25 mm i.d. ×30 m with a 0.25 µm film thickness. High purity helium gas was used as the carrier gas and maintained at a flow rate of 1 mL min^−1^. The temperature of GC injection port was set at 250 °C in the splitless mode. The GC program was set as initial temperature 70 °C, held for 3 min; ramped at 20 °C/min to 90 °C; 3 °C/min to 170 °C; 30 °C/min to 280 °C, and then 20 °C/min to 300 °C (5 min hold). For the MS detector, the temperature of transfer line, quadruple, and ion source were set at 280 °C, 150 °C, and 230 °C, respectively, with ionization potential of 70 electron volt (eV) and scan range of 50 to 350 atomic mass units. Relative distribution of the compounds was estimated based on the peak area, which was automatically integrated by a GC analytic software (MSD ChemStation E.02.02.1431; Agilent Technologies). All terpenoids detected were identified by comparing their retention times and mass spectra with those of authentic standards, or with data retrieved from the Wiley Registry (12th edition) and the 2020 NIST Mass Spectral Library (Version 2.4).

### 4.5. Total RNA Isolation and cDNA Synthesis

Shoot tissues were pulverized using a mortar and a pestle with liquid nitrogen. Total RNA was extracted using RNeasy^®^ Plant Mini Kit (Qiagen, Germantown, MD, USA), according to manufacturer’s instruction and quantified using a nanodrop (Nanodrop lite, Thermo Scientific, Waltham, MA, USA), followed by complementary DNA synthesis using LaboPassTM cDNA Synthesis Kit (Cosmo Genetech, Seoul, Republic of Korea).

### 4.6. Gene Expression Analysis of LsTPSs Transcripts

Semi-quantitative reverse transcription (RT)-polymerase chain reaction (PCR) was performed with gene-specific primers (Table S3) in a thermal program as follows: 95 °C for 3 min; 29 cycles of 95 °C for 30 s, 56 °C for 30 s, 72 °C for 30 s; and a final extension at 72 °C for 5 min. The PCR products were visualized on a 1.5% (*w*/*v*) agarose gel after staining with GelRedTM Nucleic Acid Stain (Biotium, Fremont, CA, USA). Quantitative real-time PCRs (qRT-PCRs) were performed using iTaq Universal SYBR^®^ Green Supermix (Bio-Rad Laboratories, Hercules, CA, USA) with gene-specific primers (Table S4), in a CFX ConnectTM Real-Time PCR Detection System (Bio-Rad Laboratories, Hercules, CA, USA) with the following cycles: 95 °C for 30 s, followed by 39 cycles of 95 °C for 10 s, 59 °C for 10 s and 72 °C for 30 s. The relative steady-state transcript abundance was quantified using the 2^−ΔΔCT^ method [48]. For normalizing gene expressions, *L. sativa* Tubulin (*LsTub*) [49] was used as an endogenous control. The qRT-PCR assays were confirmed in triplicate using three biological replicates with three technical replicates.

### 4.7. Cloning of MJ-Inducible LsTPS Genes

Putative N-terminal signal peptides were predicted using TargetP 2.0 [50] and the full-length *LsTPS* genes in the absence of putative signal peptides were amplified using gene-specific primers with respective restriction enzyme sites (Table S5). The amplicons were subcloned into the pJET1.2 vector (CloneJET, Thermo Fisher Scientific, Waltham, MA, USA) and the gene sequences were confirmed in both directions. The nucleotide sequences obtained in this study were mismatched with those from the Phytozome (accessed in July 2023) (Figure S2), which led to the substitution of few amino acids as shown in Supplement Figures S3–S5. For protein production in bacterial expression system, the subcloned genes were transferred to a pET28a (+) expression vector (Novagen, Madison, WI, USA) via restriction digestions generating a pET28a:*LsTPS*.

### 4.8. Heterologous Overexpression of LsTPSs in E. coli

*Escherichia coli* cell culture (2 mL) harboring the pET28a:*LsTPS* was incubated overnight in Luria–Bertani (LB) medium at 37 °C supplemented with 100 µgmL^−1^ Kanamycin. The aliquot (500 µL) from the overnight grown cells was inoculated in 500 mL LB medium with 100 µgmL^−1^ Kanamycin and further incubated at 37 °C until OD600 = 0.6–0.7, at which isopropyl-β-D-1-thiogalactopyranoside (IPTG) was added at a final concentration of 0.4 mM in the medium. After a 6 h cultivation at 28 °C at 180 rpm, the cells were collected by centrifugation at 10,000× *g* for 10 min at 4 °C. The resultant pellet was resuspended in a chilled 20 mL lysis buffer (137 mM sodium chloride (NaCl), 2.7 mM potassium chloride (KCl), 10 mM disodium hydrogen phosphate, 1.8 mM monopotassium phosphate, pH 7.4) and sonicated on ice for 15 min. After centrifugation at 12,000× *g* for 30 min at 4 °C, the resulting supernatant was further purified using a 0.2 μm sterile syringe filter (Corning, Glendale, AZ, USA) prior to affinity column purification. The lysate was equilibrated with equal amount of 10 mM imidazole buffer (20 mM sodium phosphate, 300 mM NaCl, and 10 mM imidazole, pH 7.4) and loaded onto a HisPurTM Ni-NTA resin (Thermo scientific, Waltham, MA, USA) to purify His-tagged recombinant TPS proteins. Equilibrated lysate was passed through a resin column under gravity flow and elute was collected. Protein was washed with gradient concentrations of imidazole wash buffer (20 mM sodium phosphate, 300 mM sodium chloride, 25–150 mM imidazole, pH 7.4). The recombinant protein was eluted using an elution buffer containing 250 mM imidazole. The concentration of the purified protein was estimated using the Bradford assay (Bio-Rad Laboratories, Hercules, CA, USA) with bovine serum albumin as a quantification standard. Total and purified proteins were visualized on an SDS-polyacrylamide gel (10%) with Coomassie blue staining.

### 4.9. Terpene Synthase Assay

In vitro enzyme assay (1 mL) was performed at 30 °C in a 10 mL SPME glass vial capped with a PTFE/silicon septum screw containing 5 µg purified recombinant protein and an appropriate substrate. For monoterpene synthase assay, 10 µM GPP or NPP was used as a substrate in a reaction buffer [50 mM Tris-HCl (pH 7.5), 500 mM KCl, 1 mM dithiothreitol, 1 mM MnCl_2_, and 10% (*v*/*v*) glycerol]. Sesquiterpene synthase assay was performed by using 15 µM *trans*- or *cis*-FPP as a substrate in a reaction buffer [50 mM Tris-HCl (pH 7.5), 500 mM KCl, 1 mM dithiothreitol, 10 mM MgCl_2_ and 10% (*v*/*v*) glycerol], whereas 10 µM GGPP was added in a reaction buffer [50 mM Tris-HCl (pH 7.5), 500 mM KCl, 1 mM dithiothreitol, 7.5 mM MgCl_2_, 10 mM MnCl_2_, and 10% (*v*/*v*) glycerol] for diterpene synthase assay. After 2 h incubation at 30 °C, headspace volatiles were adsorbed for 60 min with a SPME fiber and subsequently analyzed by a GC–MS.

### 4.10. Chromosomal Mapping of Putative LsTPSs

Genomic information (i.e., chromosomal location, gene length, relative distance between genes) of putative terpene synthase genes of *L. sativa* was obtained from the Phytozome. A graphical chromosome map was created by manual curation for visual enhancement.

### 4.11. Sequence Alignment and Phylogenetic Tree Analysis

The deduced full-length amino acid sequences including putative transit peptides were aligned using CLUSTALW program with default parameters and visualized using the Genedoc tool v2.7.0 [51]. A maximum-likelihood phylogenetic tree was constructed using the MEGA11 software (v11.0.13) with a default setting and bootstrap values were calculated with 1000 replications [52].

### 4.12. Statistical Analysis

Statistical analyses were performed using IBM SPSS Statistics version 31.0. Prior to analysis, all data were assessed for normal distribution and homogeneity of variance. Data for in planta terpene compounds were analyzed using one-way ANOVA followed by Tukey’s HSD test (*p* < 0.05). In contrast, qRT-PCR data were analyzed using the non-parametric Kruskal–Wallis test followed by Dunn’s post hoc test (* *p* < 0.05; ** *p* < 0.01; *** *p* < 0.001), as the data did not meet the assumptions of normality and homogeneity. All experiments were conducted using three independent biological replicates, with each replicate consisting of three plants.

## Data Availability

The datasets used and/or analyzed in the current study are available for the corresponding author upon reasonable request.

## Supplementary Materials

The following supporting information can be downloaded at: https://www.mdpi.com/article/10.3390/plants15010055/s1, Figure S1: RT-PCR analysis of putative TPS genes after MJ treatment; Figure S2: Nucleotide sequence alignment of selected lettuce TPS genes; Figure S3: Protein sequence alignment of LsTPS8; Figure S4: Protein sequence alignment of LsTPS21; Figure S5: Protein sequence alignment of LsTPS24; Figure S6: Amino acid sequence alignment of 17 MJ-induced LsTPS genes; Figure S7: Mass spectra of monoterpenes generated from TPS recombinant proteins using GPP as a substrate; Figure S8: Mass spectra of monoterpenes generated from TPS recombinant proteins using NPP as a substrate; Figure S9: Mass spectra of sesquiterpenes generated from TPS recombinant proteins using E,E-FPP as a substrate; Figure S10: Mass spectra of sesquiterpenes generated from TPS recombinant proteins using Z,Z-FPP as a substrate; Figure S11: Schematic representation of MJ responsiveness in lettuce; Figure S12: Phenotypes of MJ-treated lettuce seedlings; Table S1: A list of LsTPS in lettuce; Table S2: A list of terpene compounds identified from Figure 5, Figure 6 and Figure 7; Table S3: RT-PCR primers; Table S4: qRT-PCR primers; and Table S5: Primers for expression vector cloning.
