# Supplementary material for: Genomic Identification and Biochemical Characterization of Methyl Jasmonate (MJ)-Inducible Terpene Synthase Genes in Lettuce (Lactuca sativa L. cv. Salinas)
Source: Plants (Basel). 2025 Dec 24;15(1):55. doi: 10.3390/plants15010055 (PMC12787478; doi:10.3390/plants15010055)
Supplement: Supplementary file 1 [file plants-15-00055-s001.zip › Fig. S1. RT-PCR analysis of putative TPS genes after MJ treatment.pptx]

## Slide 1
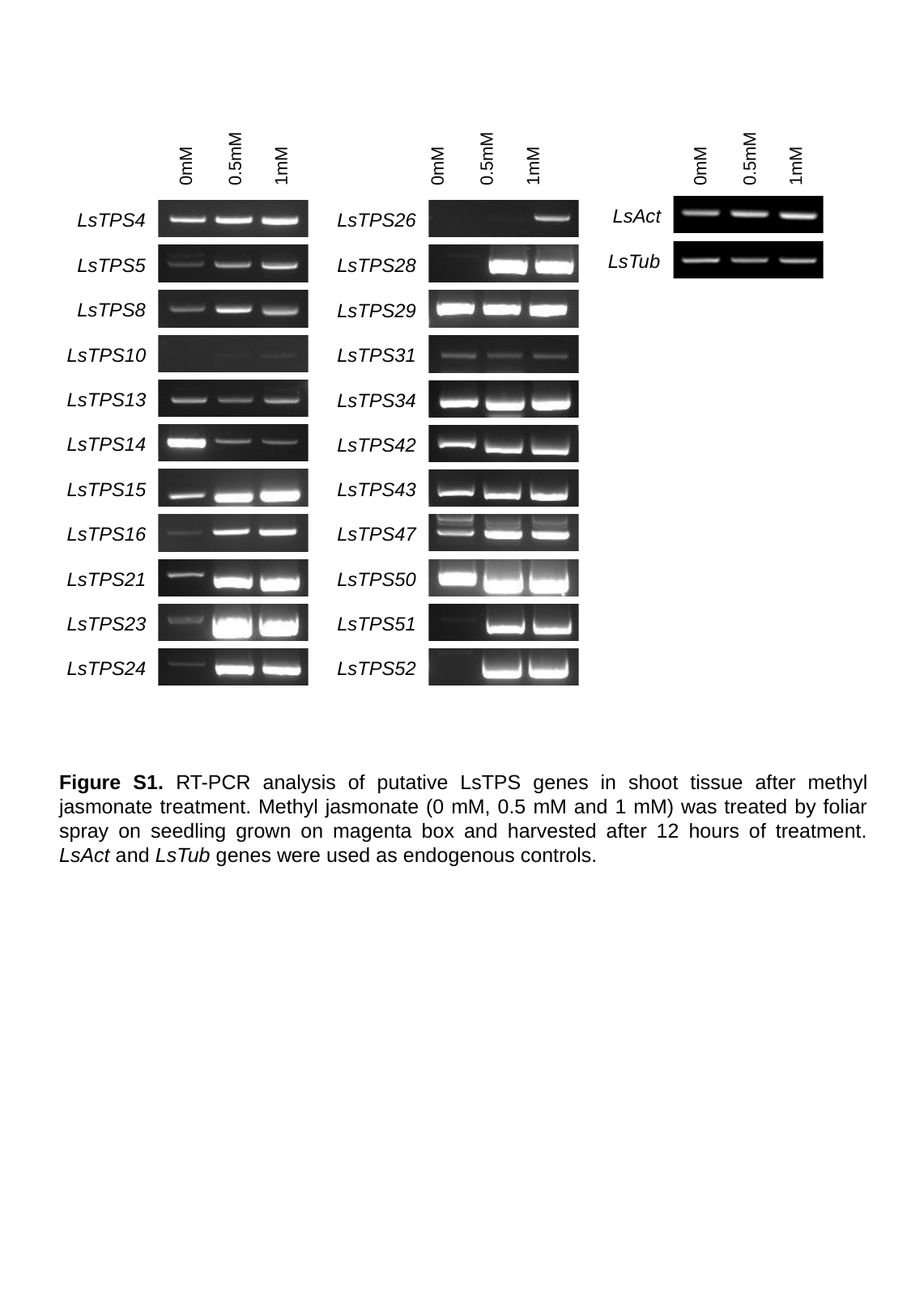

0.5mM
0mM
1mM
LsTPS4
LsTPS5
LsTPS8
LsTPS10
LsTPS13
LsTPS14
LsTPS15
LsTPS16
LsTPS21
LsTPS23
LsTPS24
0.5mM
0mM
1mM
LsTPS26
LsTPS28
LsTPS29
LsTPS31
LsTPS34
LsTPS42
LsTPS43
LsTPS47
LsTPS50
LsTPS51
LsTPS52
0.5mM
0mM
1mM
LsAct
LsTub
Figure S1. RT-PCR analysis of putative LsTPS genes in shoot tissue after methyl jasmonate treatment. Methyl jasmonate (0 mM, 0.5 mM and 1 mM) was treated by foliar spray on seedling grown on magenta box and harvested after 12 hours of treatment. LsAct and LsTub genes were used as endogenous controls.
