# Supplementary material for: Genomic Identification and Biochemical Characterization of Methyl Jasmonate (MJ)-Inducible Terpene Synthase Genes in Lettuce (Lactuca sativa L. cv. Salinas)
Source: Plants (Basel). 2025 Dec 24;15(1):55. doi: 10.3390/plants15010055 (PMC12787478; doi:10.3390/plants15010055)
Supplement: Supplementary file 1 [file plants-15-00055-s001.zip › Fig. S10. Mass spectra of sesquiterpenes generated from TPS recombinant proteins using Z,Z-FPP as a substrate.pptx]

## Slide 1
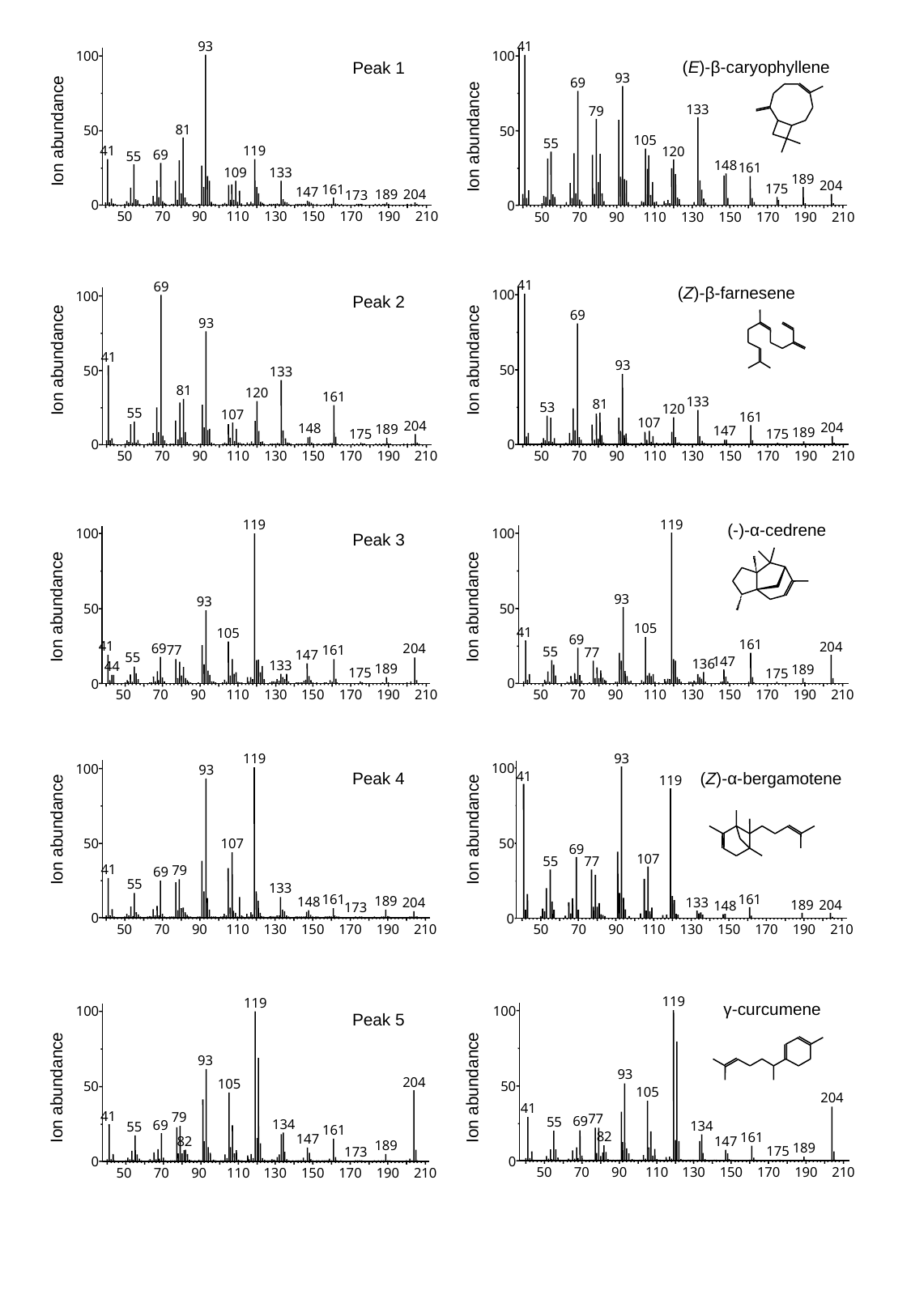

93
100
81
50
41
119
69
55
109
133
161
147
189
204
173
0
50
70
90
110
130
150
170
190
210
41
100
93
69
133
79
50
105
55
120
148
161
189
204
175
0
50
70
90
110
130
150
170
190
210
(E)-β-caryophyllene
Peak 1
Ion abundance
Ion abundance
(Z)-β-farnesene
41
100
69
93
50
133
81
53
120
161
107
204
147
189
175
0
50
70
90
110
130
150
170
190
210
69
100
93
41
50
133
81
120
161
55
107
204
148
189
175
0
50
70
90
110
130
150
170
190
210
Peak 2
Ion abundance
Ion abundance
(-)-α-cedrene
119
100
93
50
105
41
69
161
204
55
77
147
136
189
175
0
50
70
90
110
130
150
170
190
210
119
100
93
50
105
41
69
204
77
161
147
55
133
44
189
175
0
50
70
90
110
130
150
170
190
210
Peak 3
Ion abundance
Ion abundance
93
100
41
119
50
69
107
55
77
161
133
189
204
148
0
50
70
90
110
130
150
170
190
210
119
100
93
107
50
41
79
69
55
133
161
189
148
204
173
0
50
70
90
110
130
150
170
190
210
Peak 4
(Z)-α-bergamotene
Ion abundance
Ion abundance
γ-curcumene
119
100
93
50
105
204
41
77
69
55
134
82
161
147
189
175
0
50
70
90
110
130
150
170
190
210
119
100
93
204
105
50
41
79
134
69
55
161
147
82
189
173
0
50
70
90
110
130
150
170
190
210
Peak 5
Ion abundance
Ion abundance

## Slide 2
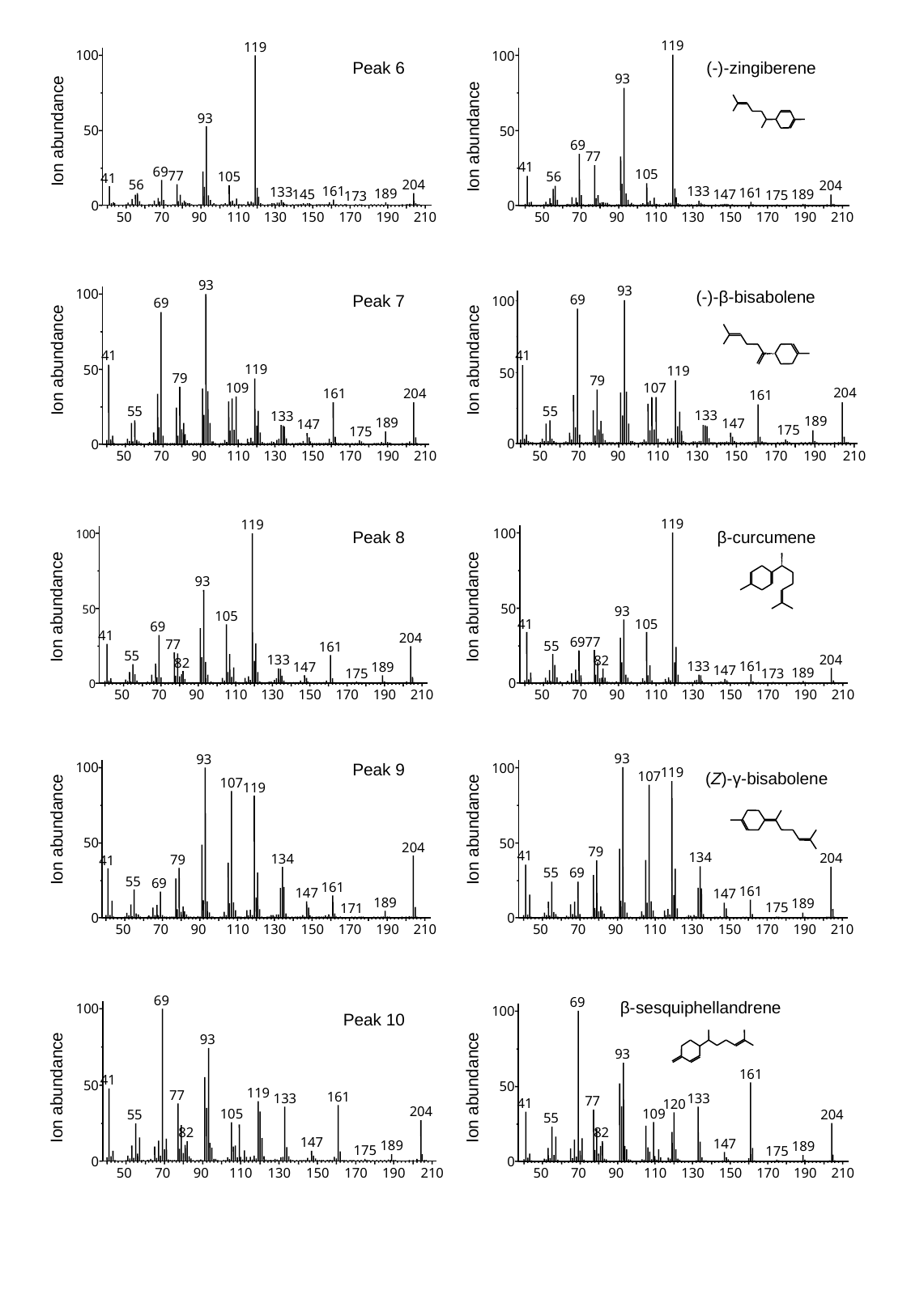

119
100
93
50
69
77
41
105
56
204
133
161
147
189
175
0
50
70
90
110
130
150
170
190
210
119
100
93
50
69
77
105
41
56
204
161
133
189
145
173
0
50
70
90
110
130
150
170
190
210
Peak 6
(-)-zingiberene
Ion abundance
Ion abundance
93
100
69
41
119
50
79
109
161
204
55
133
189
147
175
0
50
70
90
110
130
150
170
190
210
(-)-β-bisabolene
93
69
100
41
119
50
79
107
204
161
55
133
189
147
175
0
50
70
90
110
130
150
170
190
210
Peak 7
Ion abundance
Ion abundance
119
100
50
93
41
105
77
69
55
204
82
161
133
147
189
173
0
50
70
90
110
130
150
170
190
210
119
100
93
50
105
69
41
204
77
161
55
133
82
147
189
175
0
50
70
90
110
130
150
170
190
210
β-curcumene
Peak 8
Ion abundance
Ion abundance
93
100
119
107
50
79
41
134
204
55
69
161
147
189
175
0
50
70
90
110
130
150
170
190
210
93
100
107
119
50
204
134
79
41
55
69
161
147
189
171
0
50
70
90
110
130
150
170
190
210
Peak 9
(Z)-γ-bisabolene
Ion abundance
Ion abundance
β-sesquiphellandrene
69
100
93
41
50
119
77
161
133
204
105
55
82
147
189
175
0
50
70
90
110
130
150
170
190
210
69
100
93
161
50
133
77
41
120
109
204
55
82
147
189
175
0
50
70
90
110
130
150
170
190
210
Peak 10
Ion abundance
Ion abundance

## Slide 3
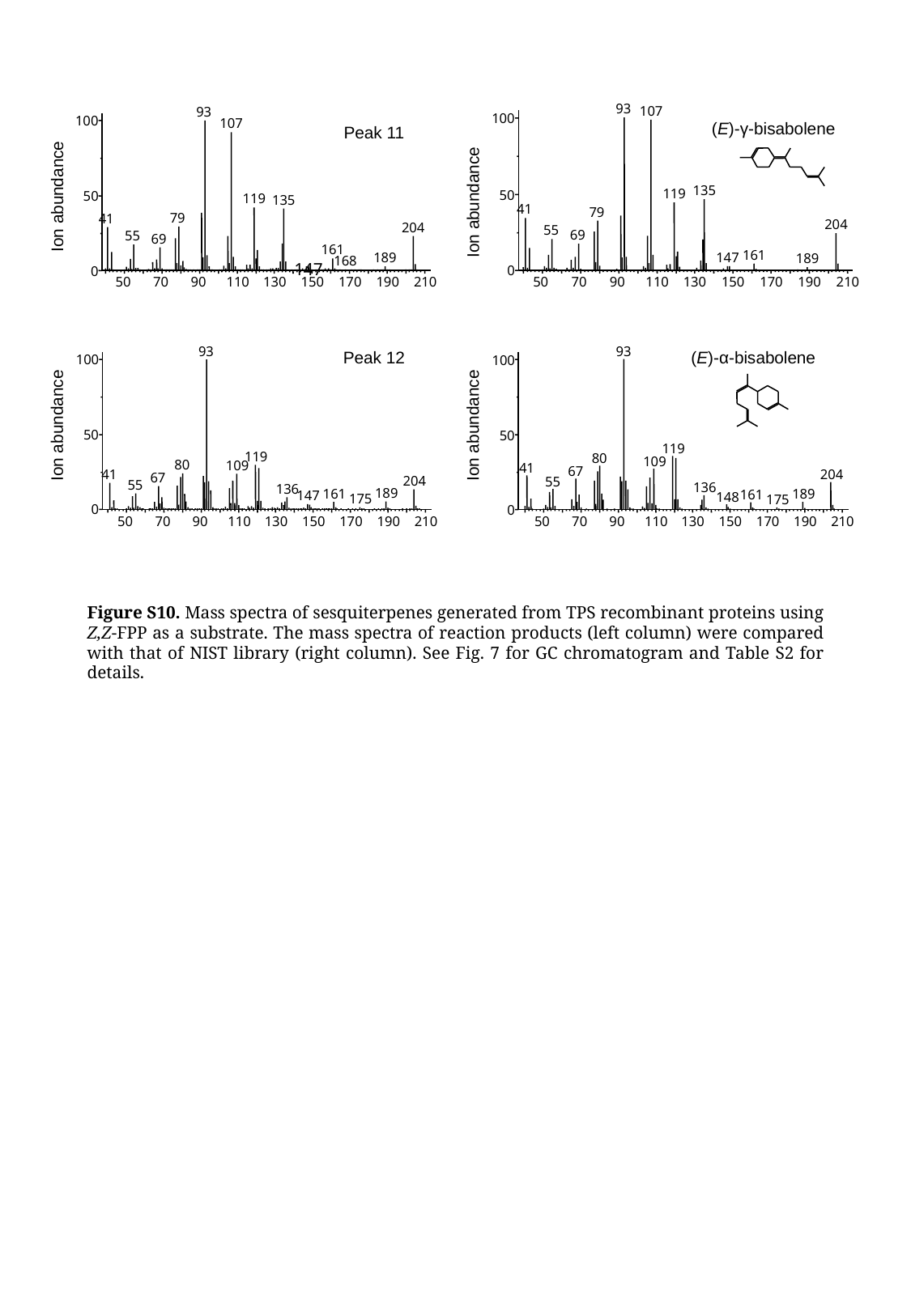

93
107
100
135
119
50
41
79
204
55
69
161
147
189
0
50
70
90
110
130
150
170
190
210
93
100
107
50
119
135
79
41
204
55
69
161
189
168
147
0
50
70
90
110
130
150
170
190
210
(E)-γ-bisabolene
Peak 11
Ion abundance
Ion abundance
Peak 12
(E)-α-bisabolene
93
100
50
119
80
109
41
67
204
55
136
189
161
147
175
0
50
70
90
110
130
150
170
190
210
93
100
50
119
80
109
41
67
204
55
136
189
161
148
175
0
50
70
90
110
130
150
170
190
210
Ion abundance
Ion abundance
Figure S10. Mass spectra of sesquiterpenes generated from TPS recombinant proteins using Z,Z-FPP as a substrate. The mass spectra of reaction products (left column) were compared with that of NIST library (right column). See Fig. 7 for GC chromatogram and Table S2 for details.
