# Supplementary material for: Genomic Identification and Biochemical Characterization of Methyl Jasmonate (MJ)-Inducible Terpene Synthase Genes in Lettuce (Lactuca sativa L. cv. Salinas)
Source: Plants (Basel). 2025 Dec 24;15(1):55. doi: 10.3390/plants15010055 (PMC12787478; doi:10.3390/plants15010055)
Supplement: Supplementary file 1 [file plants-15-00055-s001.zip › Fig. S11. Schematic representation of MJ responsiveness in lettuce.pptx]

## Slide 1
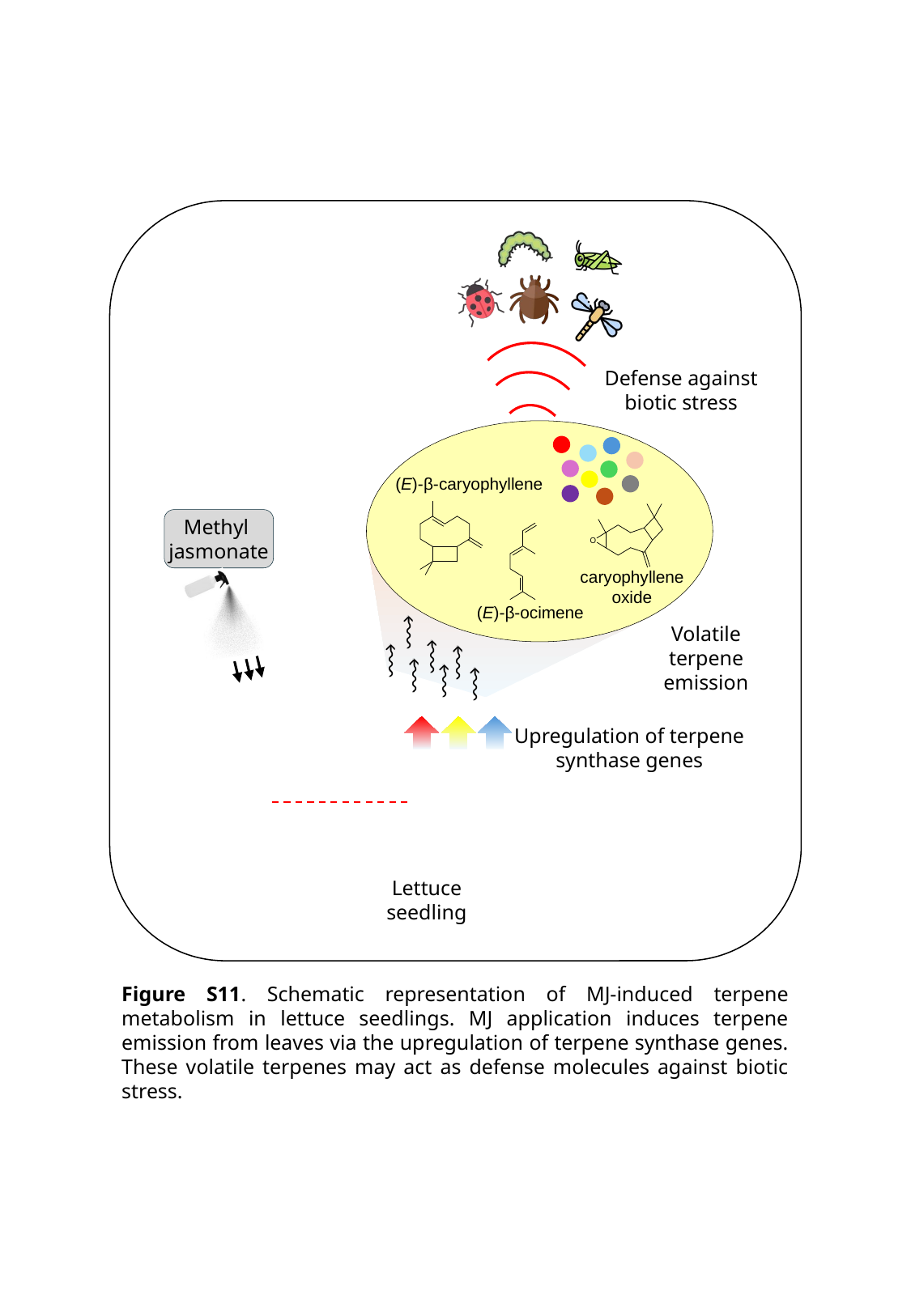

Defense against biotic stress
(E)-β-caryophyllene
caryophyllene oxide
Methyl
jasmonate
(E)-β-ocimene
Volatile terpene emission
Upregulation of terpene synthase genes
Lettuce seedling
Figure S11. Schematic representation of MJ-induced terpene metabolism in lettuce seedlings. MJ application induces terpene emission from leaves via the upregulation of terpene synthase genes. These volatile terpenes may act as defense molecules against biotic stress.
