# Supplementary material for: Genomic Identification and Biochemical Characterization of Methyl Jasmonate (MJ)-Inducible Terpene Synthase Genes in Lettuce (Lactuca sativa L. cv. Salinas)
Source: Plants (Basel). 2025 Dec 24;15(1):55. doi: 10.3390/plants15010055 (PMC12787478; doi:10.3390/plants15010055)
Supplement: Supplementary file 1 [file plants-15-00055-s001.zip › Fig. S12. Phenotypes of MJ-treated lettuce seedlings.pptx]

## Slide 1
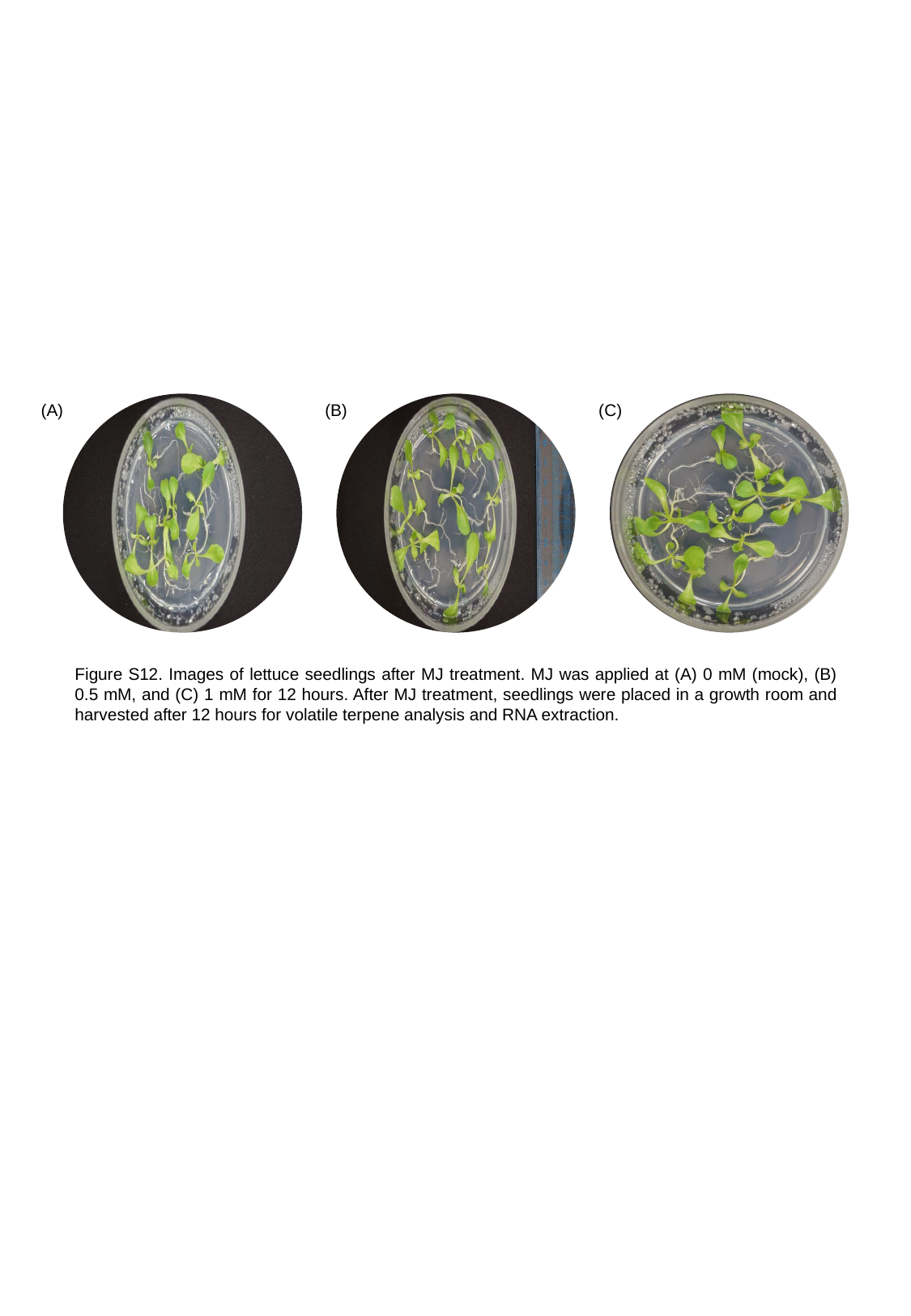

(C)
(A)
(B)
Figure S12. Images of lettuce seedlings after MJ treatment. MJ was applied at (A) 0 mM (mock), (B) 0.5 mM, and (C) 1 mM for 12 hours. After MJ treatment, seedlings were placed in a growth room and harvested after 12 hours for volatile terpene analysis and RNA extraction.
