# Supplementary material for: Genomic Identification and Biochemical Characterization of Methyl Jasmonate (MJ)-Inducible Terpene Synthase Genes in Lettuce (Lactuca sativa L. cv. Salinas)
Source: Plants (Basel). 2025 Dec 24;15(1):55. doi: 10.3390/plants15010055 (PMC12787478; doi:10.3390/plants15010055)
Supplement: Supplementary file 1 [file plants-15-00055-s001.zip › Fig. S2. Nucleotide sequence alignment of selected lettuce TPS genes.pptx]

## Slide 1
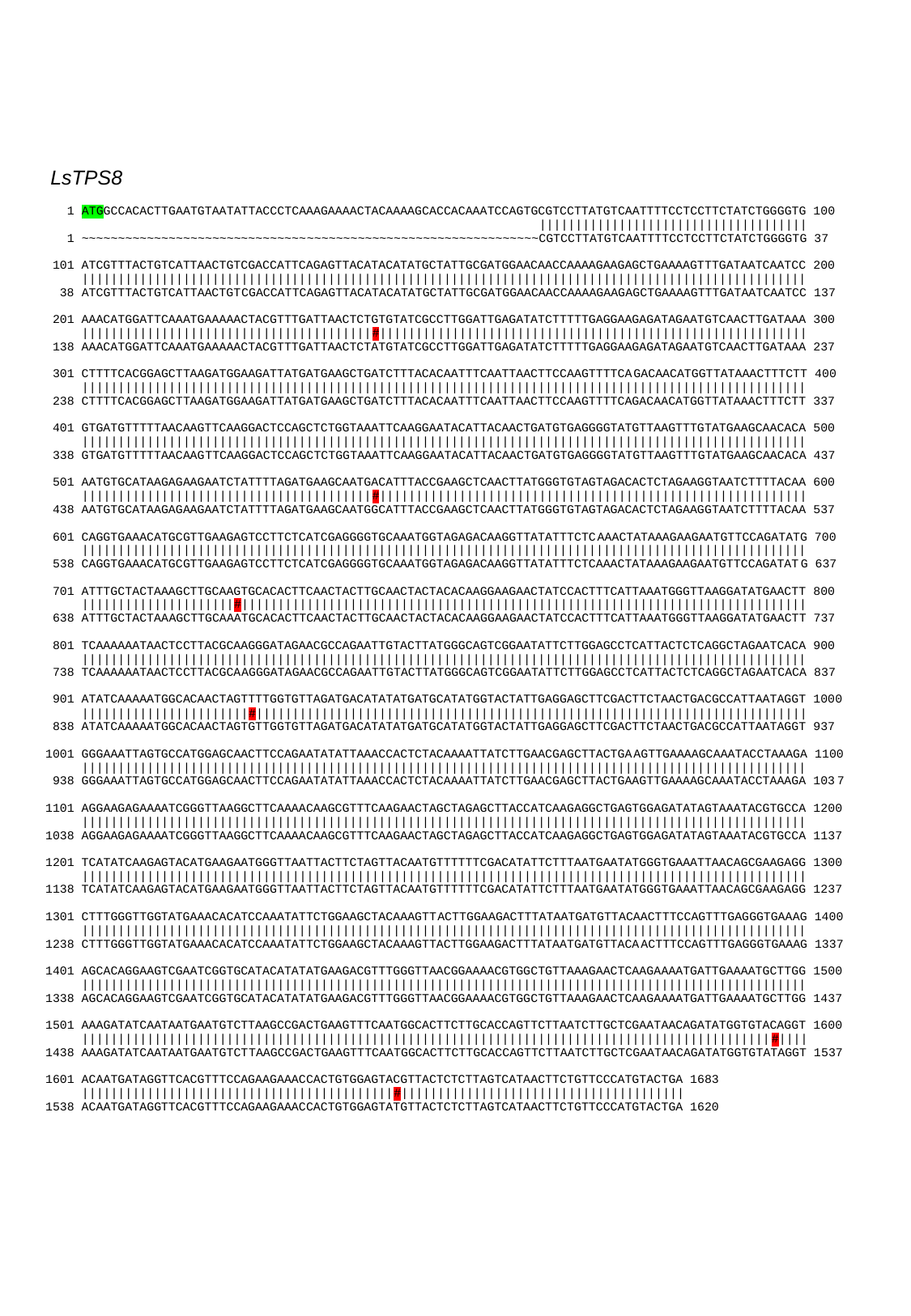

LsTPS8

## Slide 2
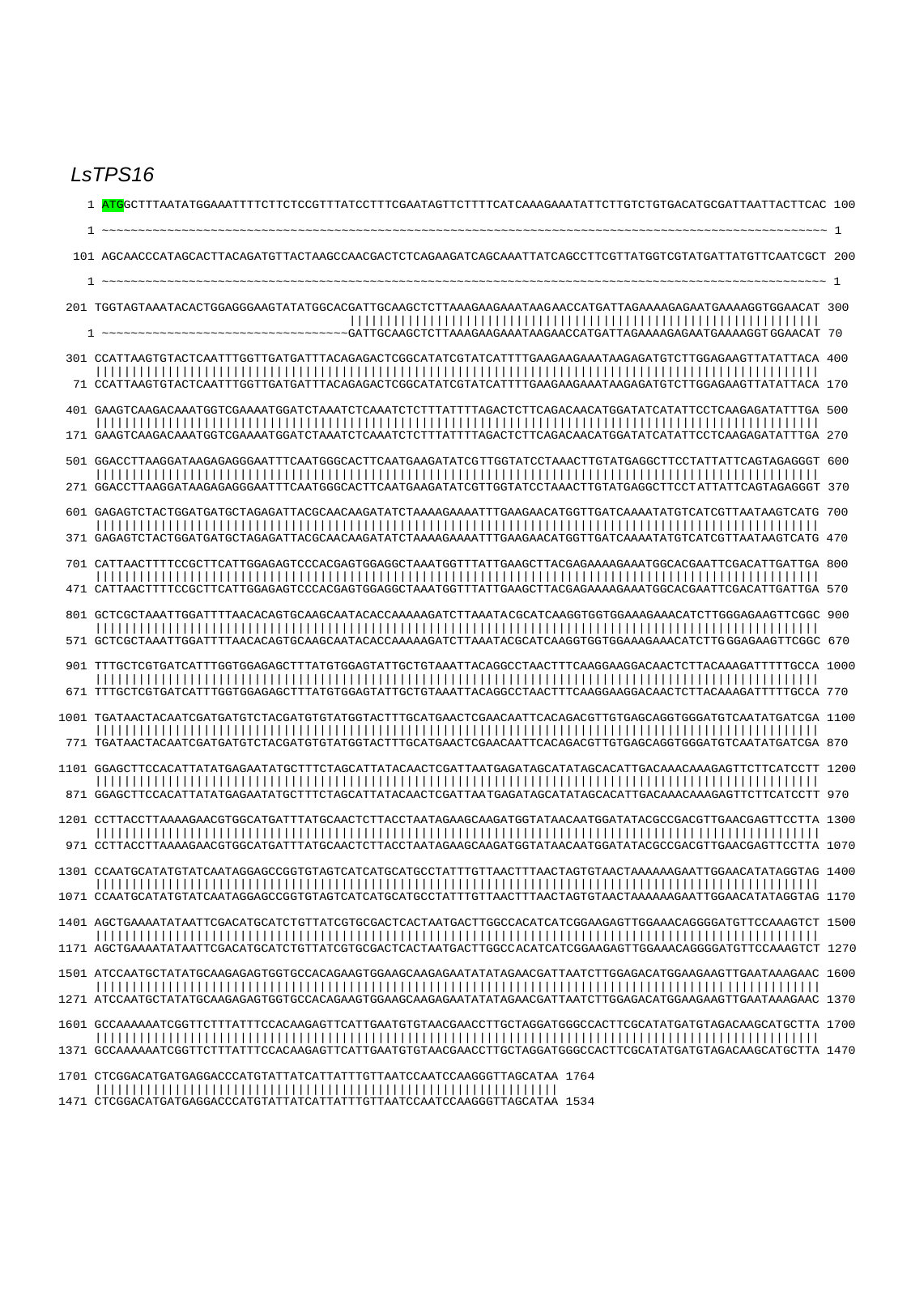

LsTPS16

## Slide 3
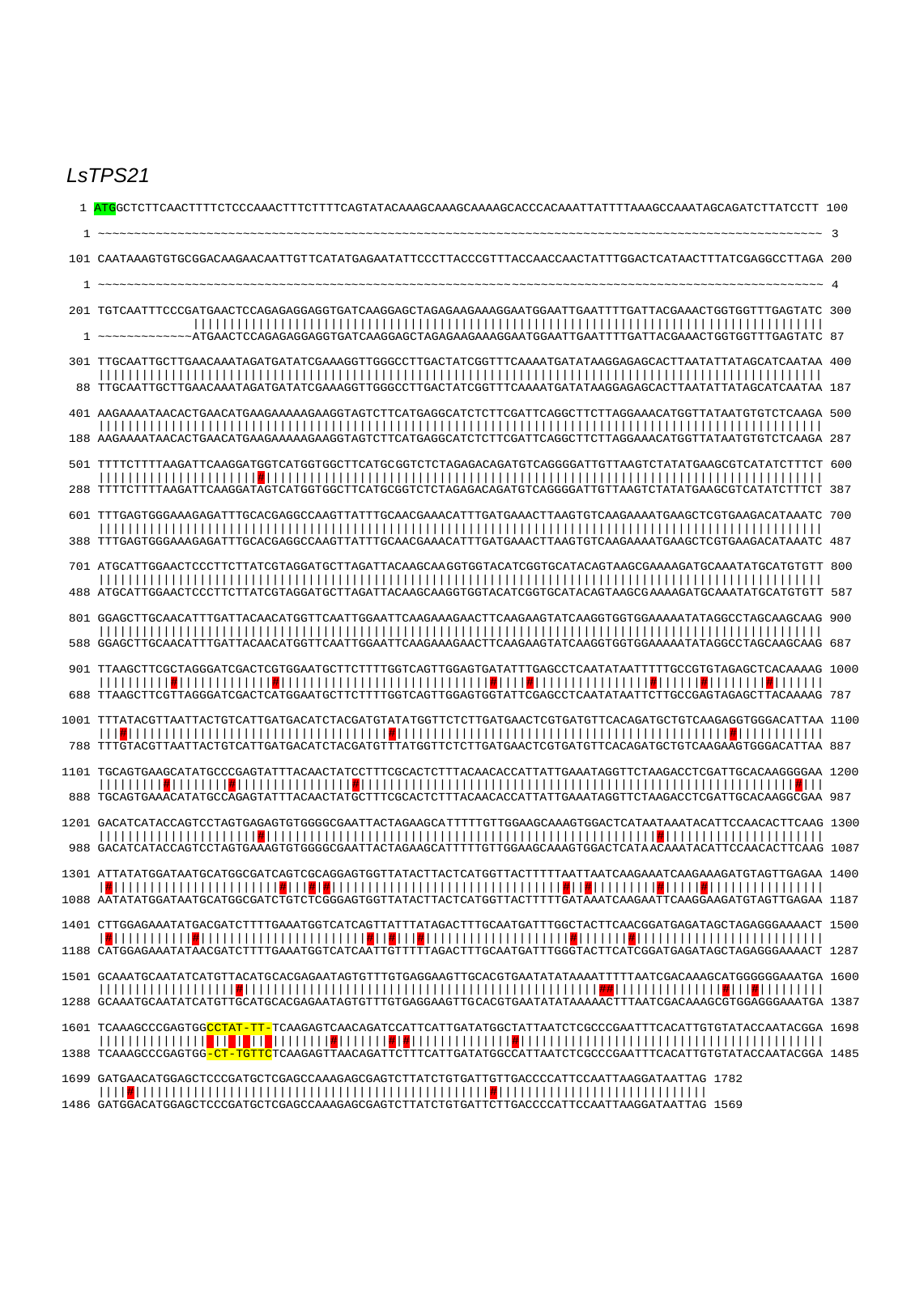

LsTPS21

## Slide 4
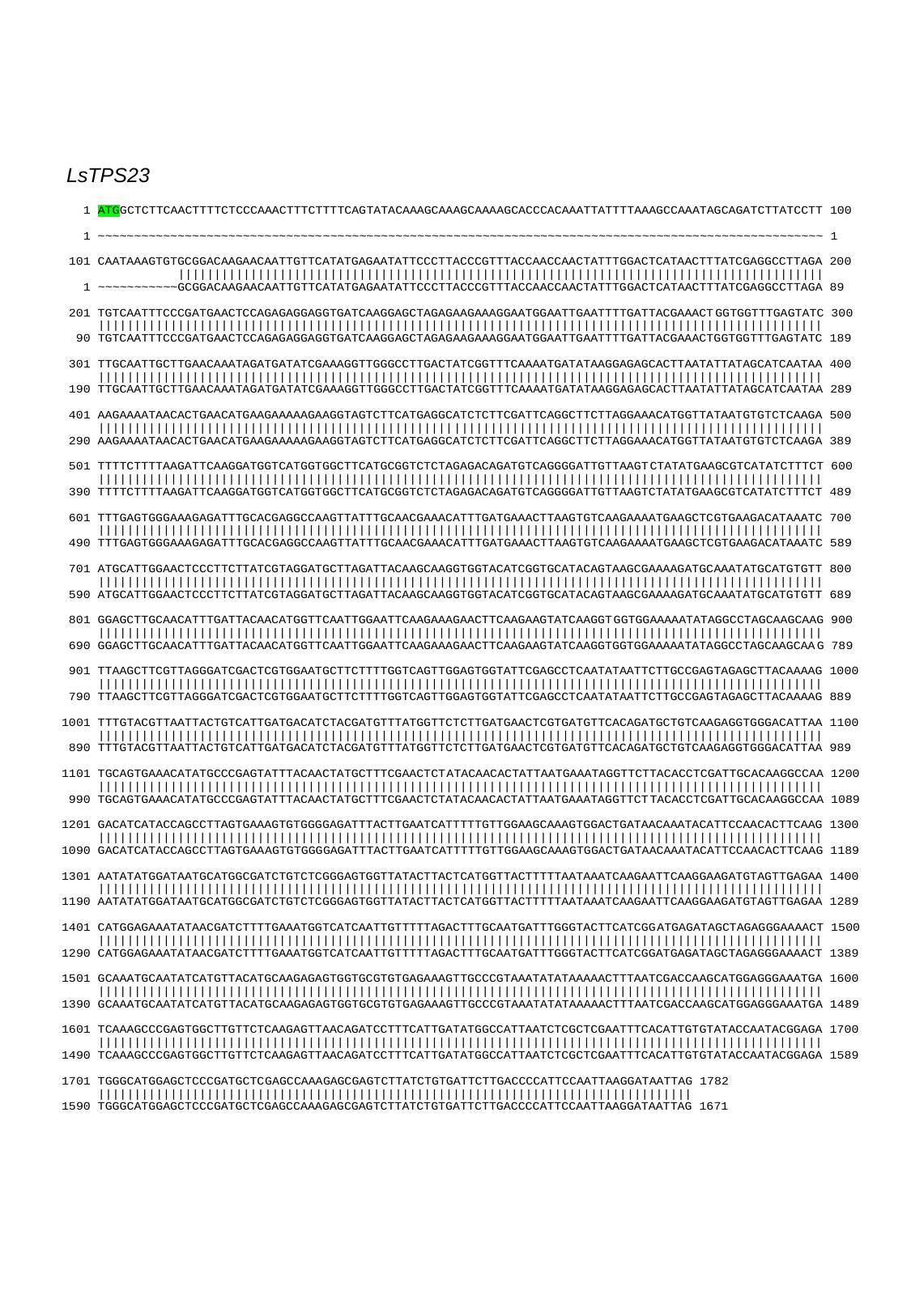

LsTPS23

## Slide 5
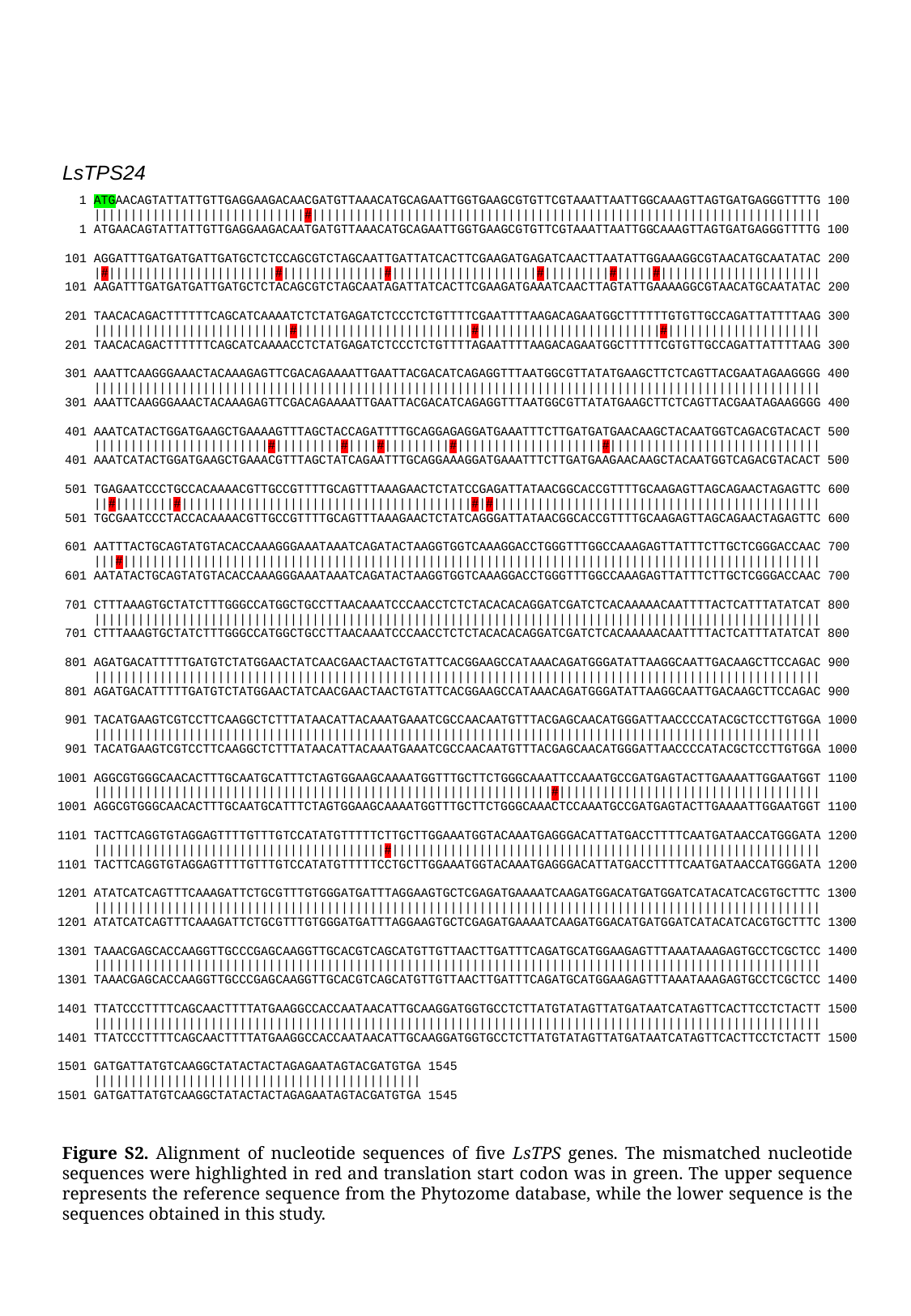

LsTPS24
Figure S2. Alignment of nucleotide sequences of five LsTPS genes. The mismatched nucleotide sequences were highlighted in red and translation start codon was in green. The upper sequence represents the reference sequence from the Phytozome database, while the lower sequence is the sequences obtained in this study.
