# Supplementary material for: Genomic Identification and Biochemical Characterization of Methyl Jasmonate (MJ)-Inducible Terpene Synthase Genes in Lettuce (Lactuca sativa L. cv. Salinas)
Source: Plants (Basel). 2025 Dec 24;15(1):55. doi: 10.3390/plants15010055 (PMC12787478; doi:10.3390/plants15010055)
Supplement: Supplementary file 1 [file plants-15-00055-s001.zip › Fig. S5. Protein sequence alignment of LsTPS24.pptx]

## Slide 1
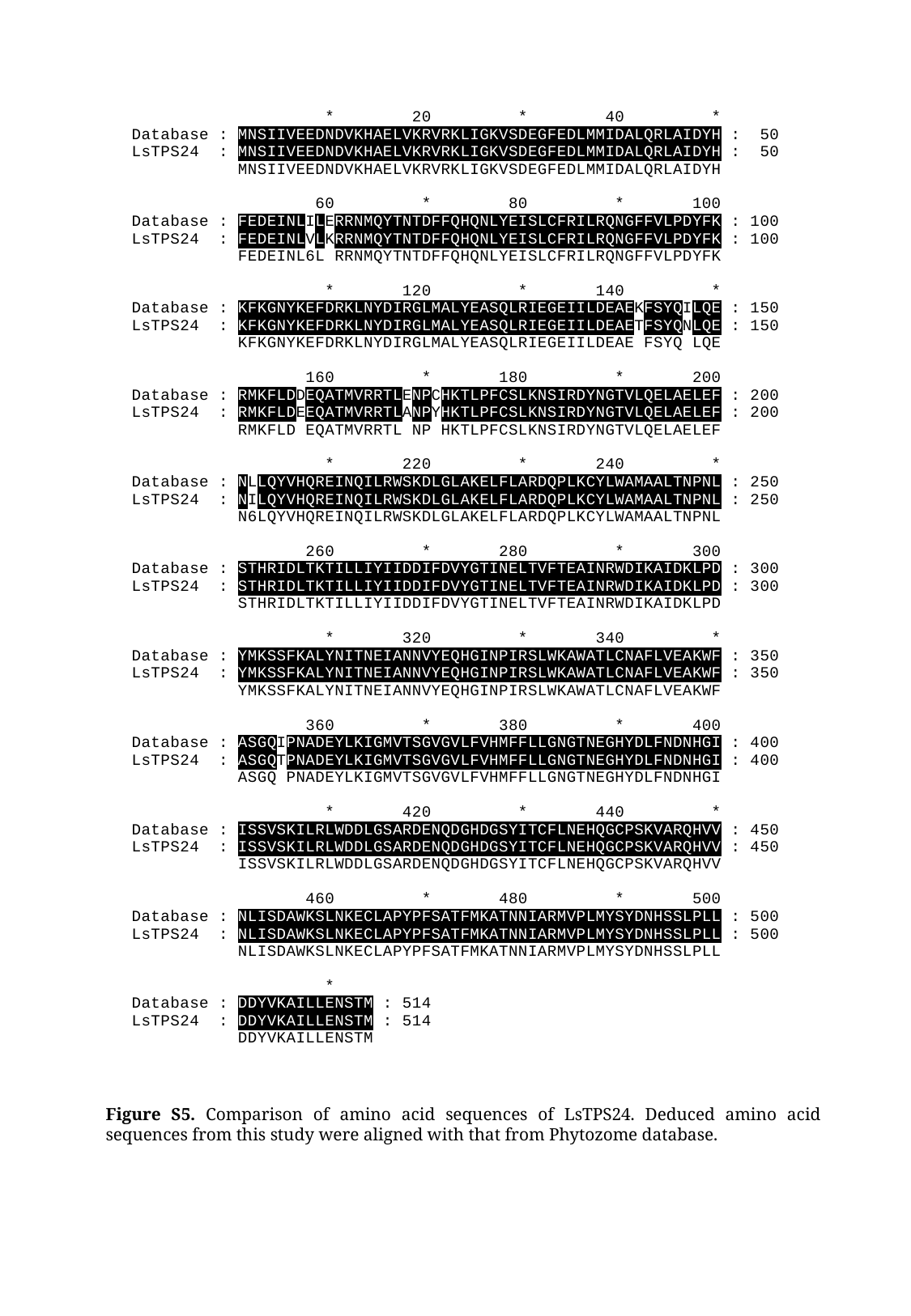

Figure S5. Comparison of amino acid sequences of LsTPS24. Deduced amino acid sequences from this study were aligned with that from Phytozome database.
