# Supplementary material for: Genomic Identification and Biochemical Characterization of Methyl Jasmonate (MJ)-Inducible Terpene Synthase Genes in Lettuce (Lactuca sativa L. cv. Salinas)
Source: Plants (Basel). 2025 Dec 24;15(1):55. doi: 10.3390/plants15010055 (PMC12787478; doi:10.3390/plants15010055)
Supplement: Supplementary file 1 [file plants-15-00055-s001.zip › Fig. S6. Amino acid sequence alignment of 17 MJ-induced LsTPS genes. .pptx]

## Slide 1
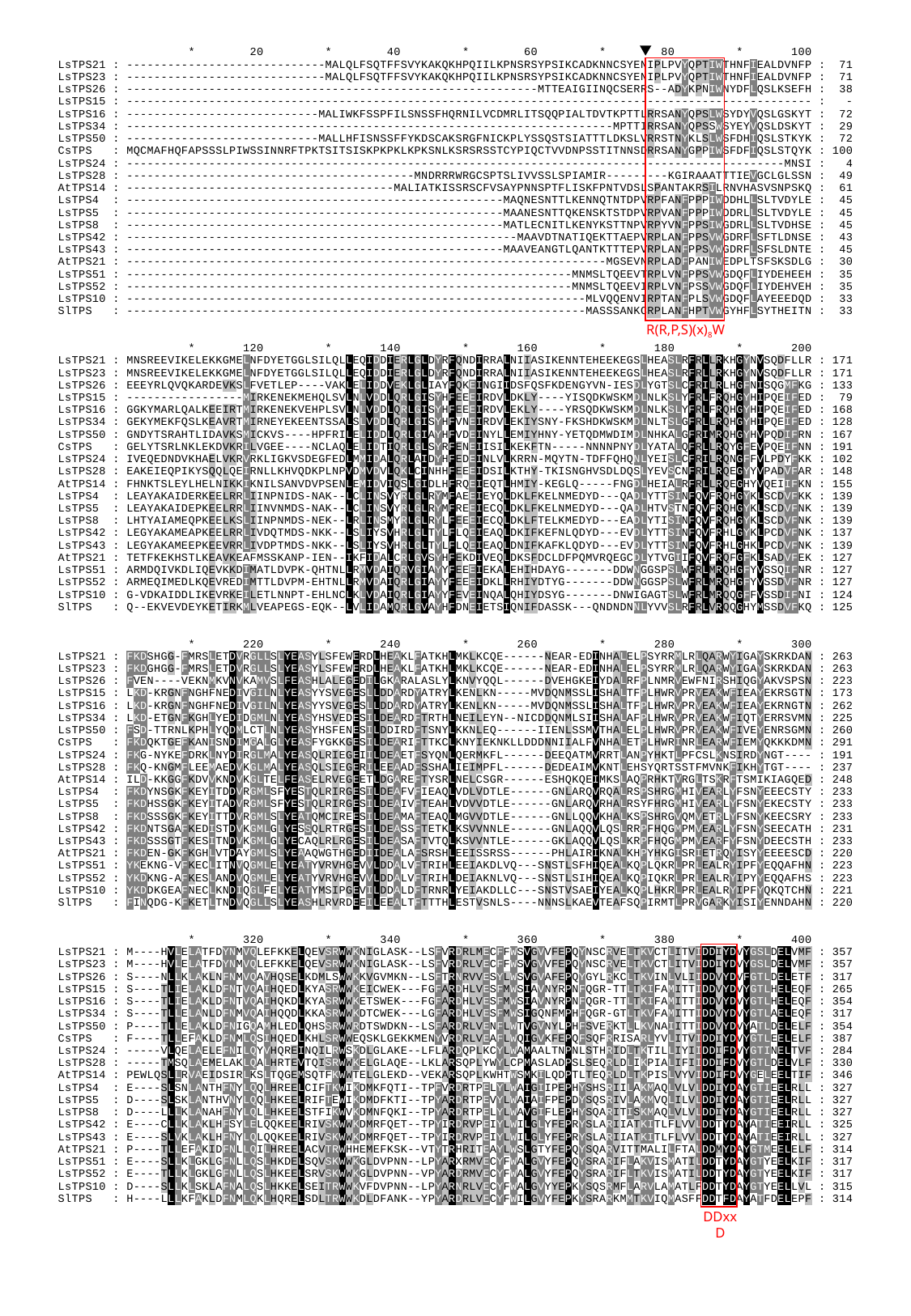

R(R,P,S)(x)8W
DDxxD

## Slide 2
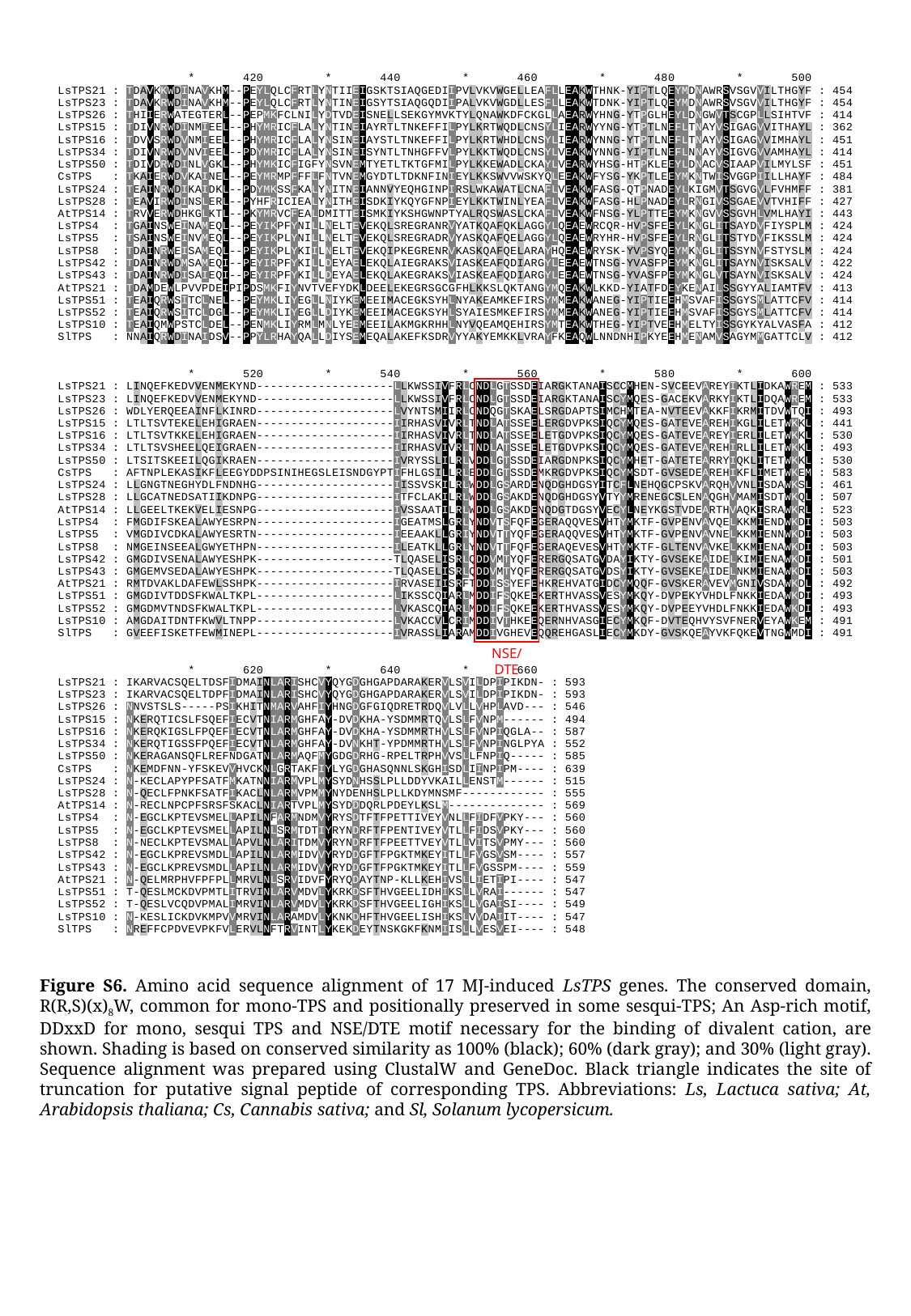

NSE/DTE
Figure S6. Amino acid sequence alignment of 17 MJ-induced LsTPS genes. The conserved domain, R(R,S)(x)8W, common for mono-TPS and positionally preserved in some sesqui-TPS; An Asp-rich motif, DDxxD for mono, sesqui TPS and NSE/DTE motif necessary for the binding of divalent cation, are shown. Shading is based on conserved similarity as 100% (black); 60% (dark gray); and 30% (light gray). Sequence alignment was prepared using ClustalW and GeneDoc. Black triangle indicates the site of truncation for putative signal peptide of corresponding TPS. Abbreviations: Ls, Lactuca sativa; At, Arabidopsis thaliana; Cs, Cannabis sativa; and Sl, Solanum lycopersicum.
