# Supplementary material for: Genomic Identification and Biochemical Characterization of Methyl Jasmonate (MJ)-Inducible Terpene Synthase Genes in Lettuce (Lactuca sativa L. cv. Salinas)
Source: Plants (Basel). 2025 Dec 24;15(1):55. doi: 10.3390/plants15010055 (PMC12787478; doi:10.3390/plants15010055)
Supplement: Supplementary file 1 [file plants-15-00055-s001.zip › Fig. S7. Mass spectra of monoterpenes generated from TPS recombinant proteins using GPP as a substrate.pptx]

## Slide 1
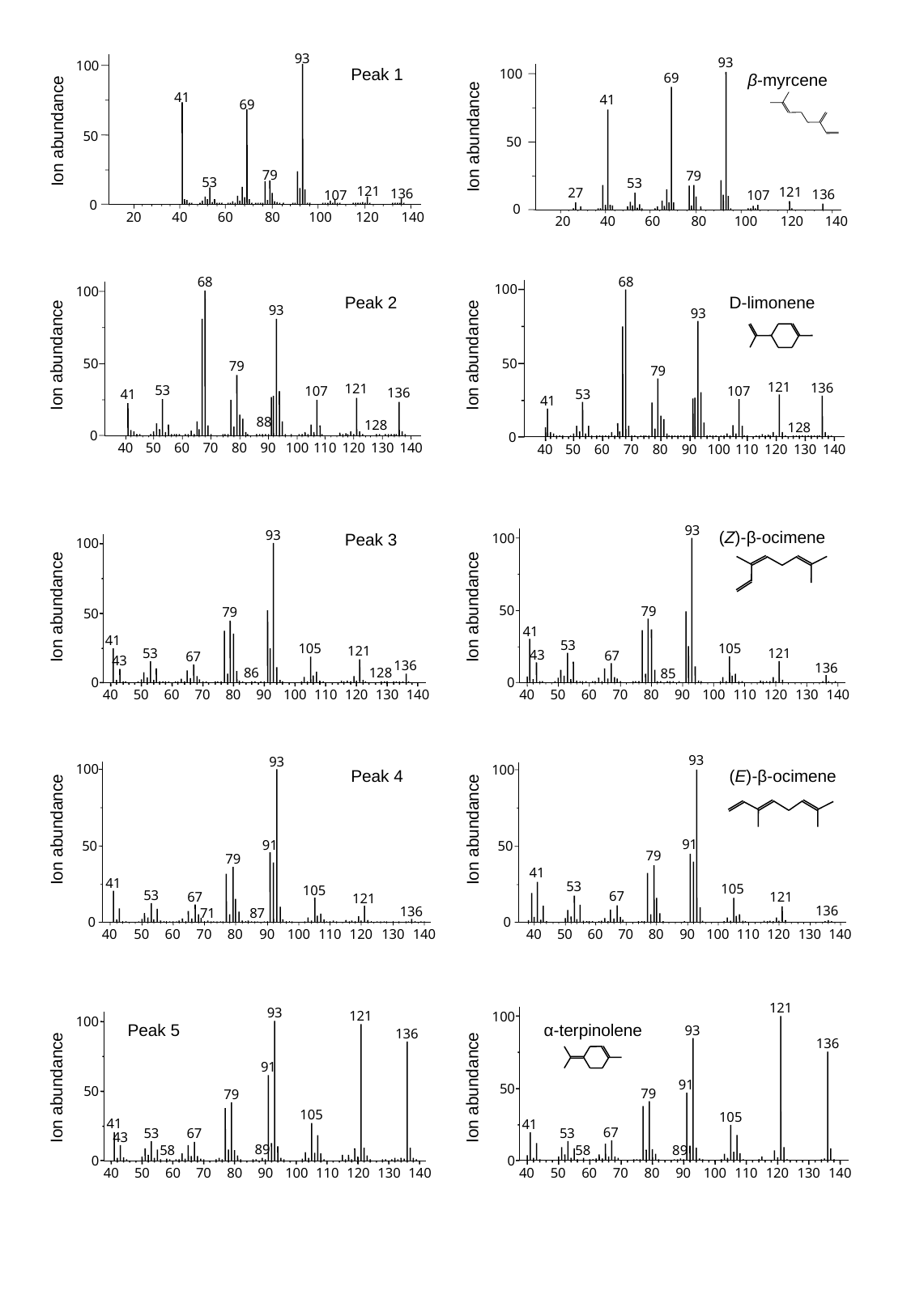

93
100
41
69
50
79
53
121
136
107
0
20
40
60
80
100
120
140
93
100
69
41
50
79
53
121
27
136
107
0
20
40
60
80
100
120
140
β-myrcene
Peak 1
Ion abundance
Ion abundance
68
100
93
50
79
121
53
107
136
41
88
128
0
40
50
60
70
80
90
100
110
120
130
140
68
100
93
50
79
121
136
107
53
41
128
0
40
50
60
70
80
90
100
110
120
130
140
Peak 2
D-limonene
Ion abundance
Ion abundance
(Z)-β-ocimene
93
100
50
79
41
53
105
121
43
67
136
85
0
40
50
60
70
80
90
100
110
120
130
140
Peak 3
93
100
79
50
41
105
121
53
67
43
136
86
128
0
40
50
60
70
80
90
100
110
120
130
140
Ion abundance
Ion abundance
93
100
91
50
79
41
53
105
67
121
136
0
40
50
60
70
80
90
100
110
120
130
140
93
100
91
50
79
41
105
53
67
121
136
71
87
0
40
50
60
70
80
90
100
110
120
130
140
Peak 4
(E)-β-ocimene
Ion abundance
Ion abundance
121
100
93
136
91
50
79
105
41
67
53
58
89
0
40
50
60
70
80
90
100
110
120
130
140
93
121
100
136
91
50
79
105
41
53
67
43
89
58
0
40
50
60
70
80
90
100
110
120
130
140
Peak 5
α-terpinolene
Ion abundance
Ion abundance

## Slide 2
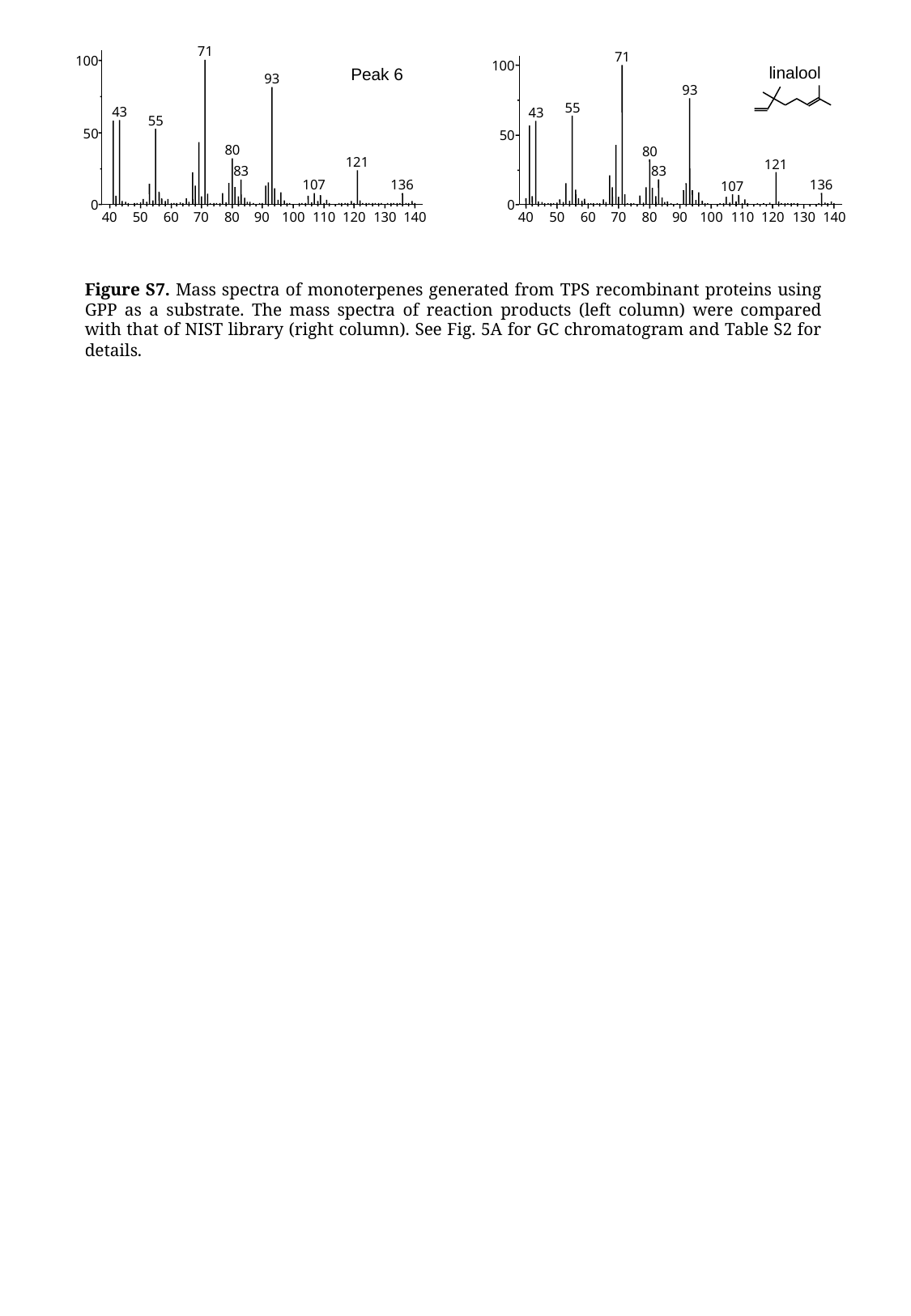

71
100
93
43
55
50
80
121
83
107
136
0
40
50
60
70
80
90
100
110
120
130
140
71
100
93
55
43
50
80
121
83
136
107
0
40
50
60
70
80
90
100
110
120
130
140
linalool
Peak 6
Figure S7. Mass spectra of monoterpenes generated from TPS recombinant proteins using GPP as a substrate. The mass spectra of reaction products (left column) were compared with that of NIST library (right column). See Fig. 5A for GC chromatogram and Table S2 for details.
