# Supplementary material for: Genomic Identification and Biochemical Characterization of Methyl Jasmonate (MJ)-Inducible Terpene Synthase Genes in Lettuce (Lactuca sativa L. cv. Salinas)
Source: Plants (Basel). 2025 Dec 24;15(1):55. doi: 10.3390/plants15010055 (PMC12787478; doi:10.3390/plants15010055)
Supplement: Supplementary file 1 [file plants-15-00055-s001.zip › Fig. S8. Mass spectra of monoterpenes generated from TPS recombinant proteins using NPP as a substrate.pptx]

## Slide 1
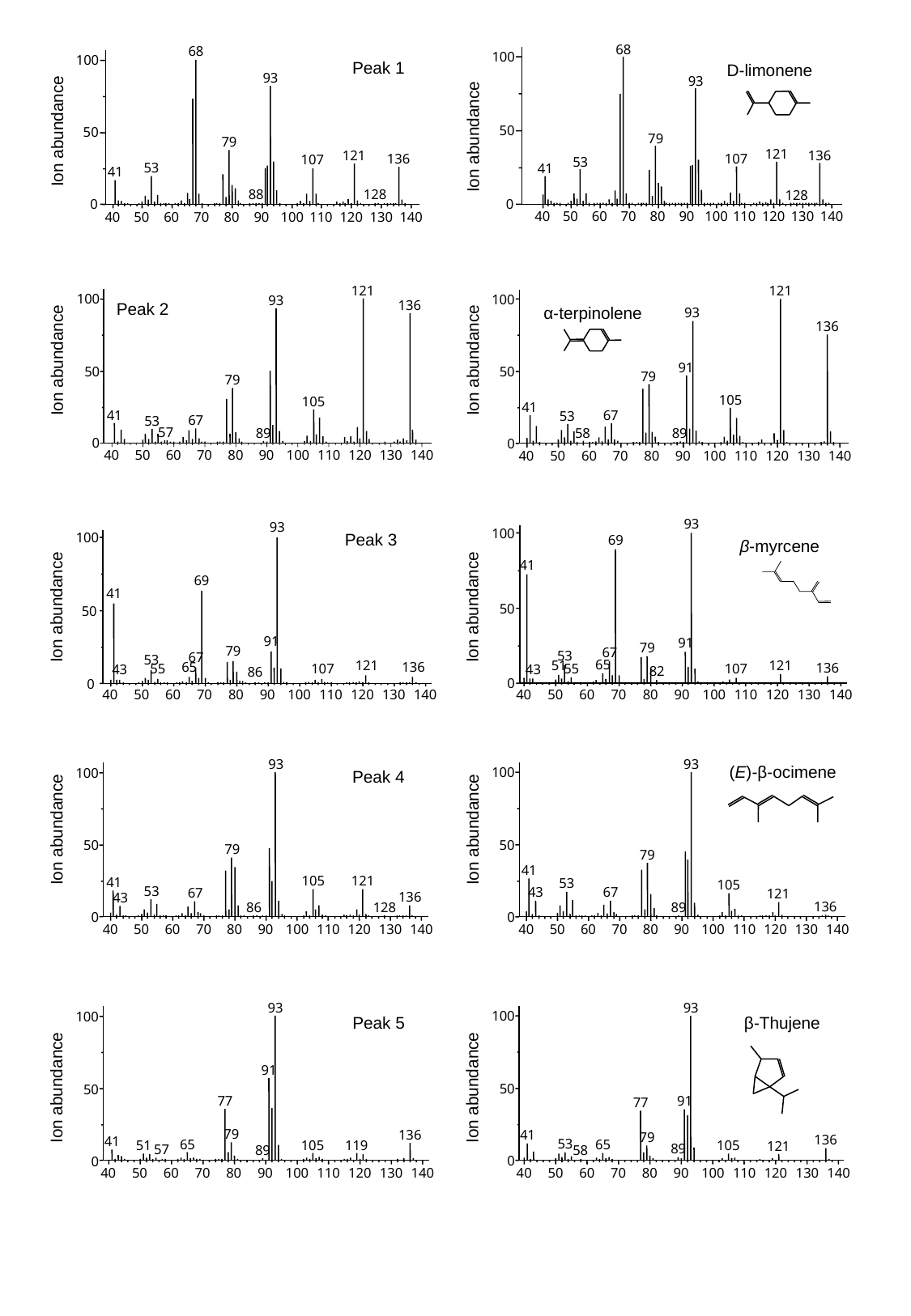

68
100
93
50
79
121
136
107
53
41
128
0
40
50
60
70
80
90
100
110
120
130
140
D-limonene
68
100
93
50
79
121
136
107
53
41
128
88
0
40
50
60
70
80
90
100
110
120
130
140
Peak 1
Ion abundance
Ion abundance
121
100
93
136
50
79
105
41
67
53
57
89
0
40
50
60
70
80
90
100
110
120
130
140
121
100
93
136
91
50
79
105
41
67
53
58
89
0
40
50
60
70
80
90
100
110
120
130
140
α-terpinolene
Peak 2
Ion abundance
Ion abundance
93
100
69
41
50
91
79
67
53
65
121
51
136
55
107
43
82
0
40
50
60
70
80
90
100
110
120
130
140
93
100
69
41
50
91
79
67
53
121
65
136
55
107
43
86
0
40
50
60
70
80
90
100
110
120
130
140
Peak 3
β-myrcene
Ion abundance
Ion abundance
(E)-β-ocimene
93
100
50
79
105
121
41
53
67
136
43
86
128
0
40
50
60
70
80
90
100
110
120
130
140
Peak 4
93
100
50
79
41
53
105
43
67
121
136
89
0
40
50
60
70
80
90
100
110
120
130
140
Ion abundance
Ion abundance
93
100
50
91
77
41
79
136
53
65
105
121
89
58
0
40
50
60
70
80
90
100
110
120
130
140
β-Thujene
93
100
91
50
77
79
136
41
65
105
119
51
57
89
0
40
50
60
70
80
90
100
110
120
130
140
Peak 5
Ion abundance
Ion abundance

## Slide 2
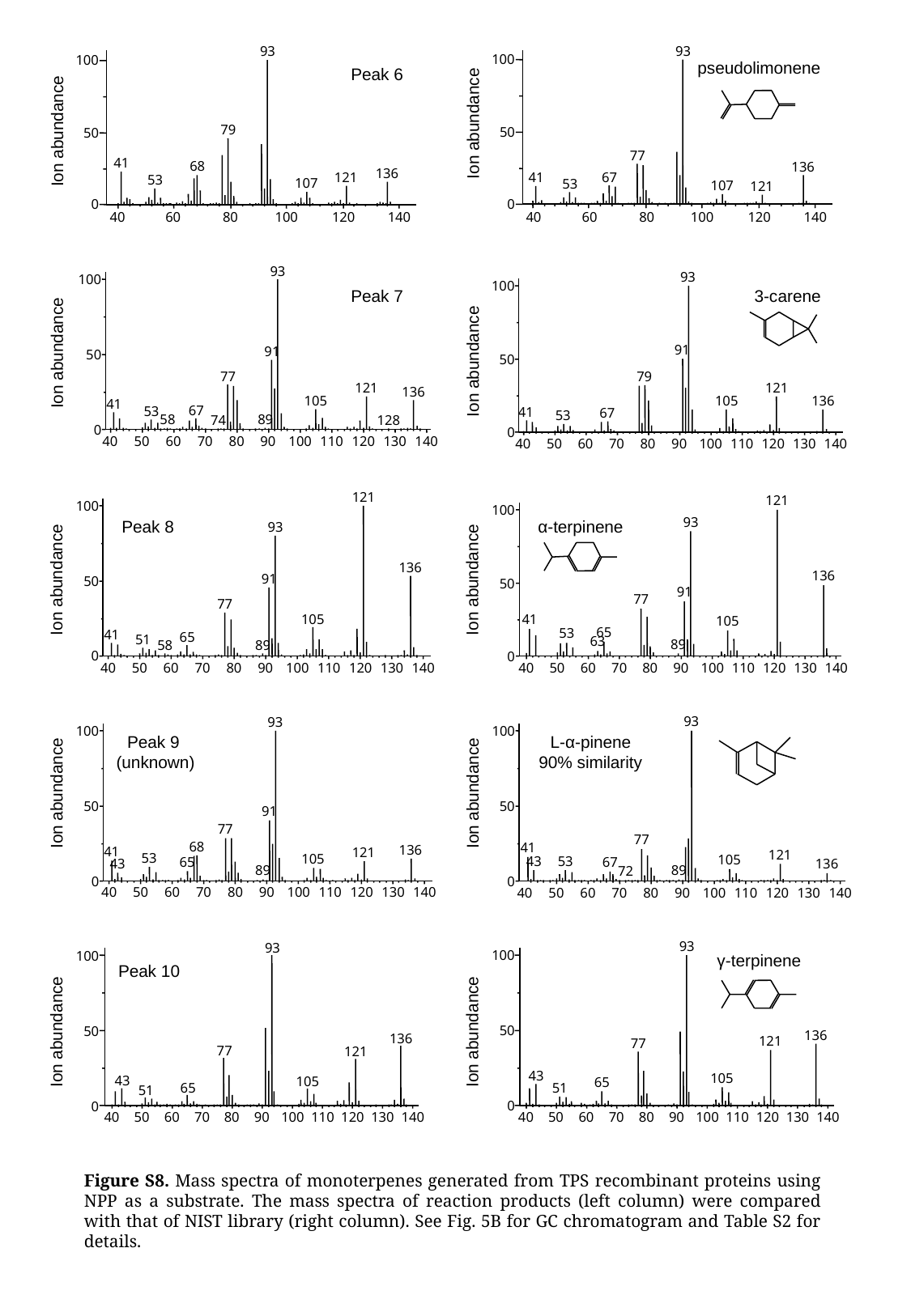

93
100
50
77
136
67
41
53
107
121
0
40
60
80
100
120
140
pseudolimonene
93
100
79
50
41
68
136
121
53
107
0
40
60
80
100
120
140
Peak 6
Ion abundance
Ion abundance
93
100
91
50
77
121
136
105
41
67
53
58
89
74
128
0
40
50
60
70
80
90
100
110
120
130
140
93
100
91
50
79
121
105
136
41
67
53
0
40
50
60
70
80
90
100
110
120
130
140
Peak 7
3-carene
Ion abundance
Ion abundance
121
100
93
136
91
50
77
105
41
65
51
58
89
0
40
50
60
70
80
90
100
110
120
130
140
121
100
93
136
50
91
77
41
105
65
53
63
89
0
40
50
60
70
80
90
100
110
120
130
140
Peak 8
α-terpinene
Ion abundance
Ion abundance
93
100
50
77
41
121
105
43
53
67
136
89
72
0
40
50
60
70
80
90
100
110
120
130
140
93
100
50
91
77
68
136
41
121
53
105
65
43
89
0
40
50
60
70
80
90
100
110
120
130
140
Peak 9
(unknown)
L-α-pinene
90% similarity
Ion abundance
Ion abundance
93
100
50
136
121
77
43
105
65
51
0
40
50
60
70
80
90
100
110
120
130
140
93
100
50
136
77
121
43
105
65
51
0
40
50
60
70
80
90
100
110
120
130
140
γ-terpinene
Peak 10
Ion abundance
Ion abundance
Figure S8. Mass spectra of monoterpenes generated from TPS recombinant proteins using NPP as a substrate. The mass spectra of reaction products (left column) were compared with that of NIST library (right column). See Fig. 5B for GC chromatogram and Table S2 for details.
