# Supplementary material for: Genomic Identification and Biochemical Characterization of Methyl Jasmonate (MJ)-Inducible Terpene Synthase Genes in Lettuce (Lactuca sativa L. cv. Salinas)
Source: Plants (Basel). 2025 Dec 24;15(1):55. doi: 10.3390/plants15010055 (PMC12787478; doi:10.3390/plants15010055)
Supplement: Supplementary file 1 [file plants-15-00055-s001.zip › Fig. S9. Mass spectra of sesquiterpenes generated from TPS recombinant proteins using E,E-FPP as a substrate.pptx]

## Slide 1
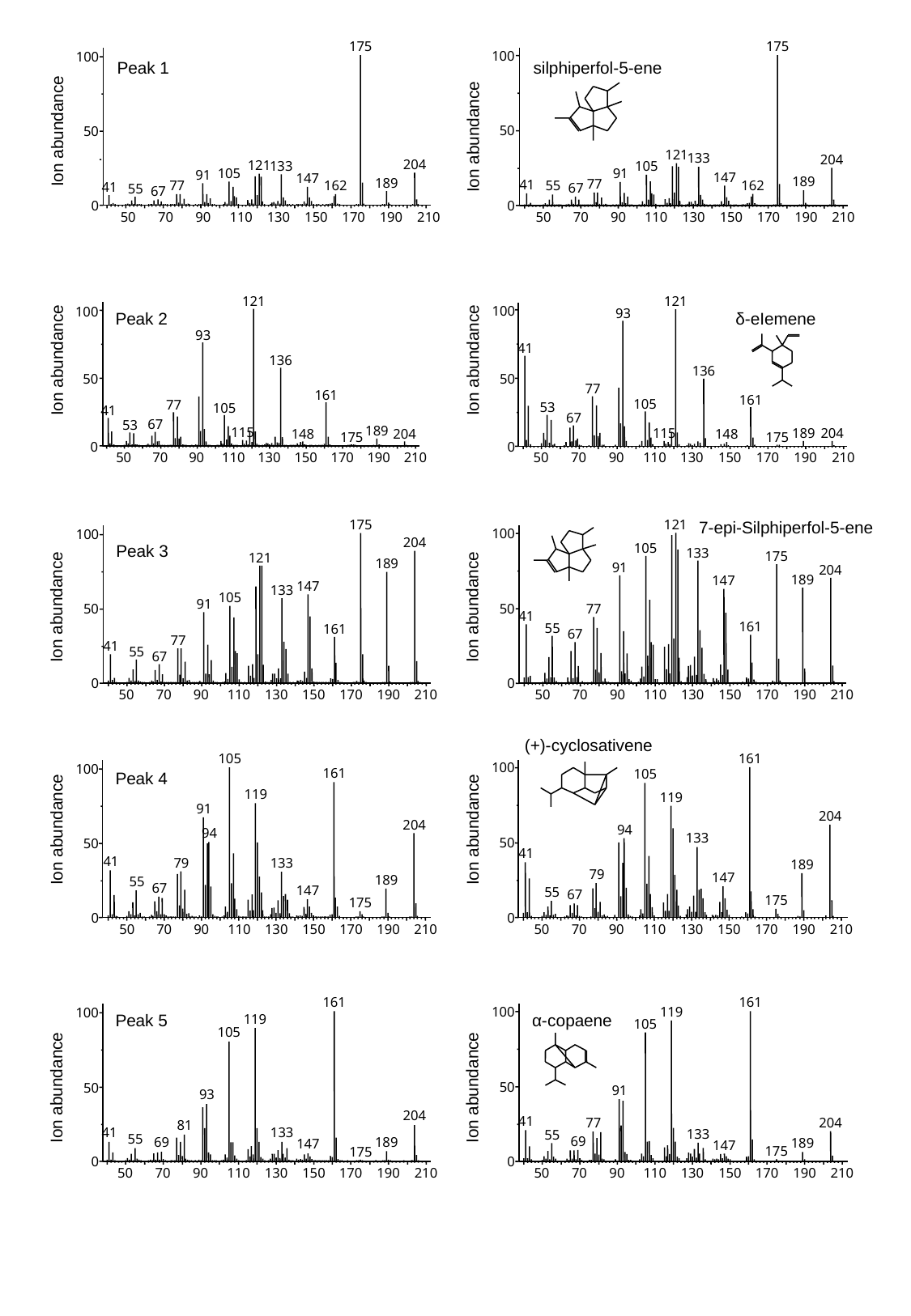

175
100
silphiperfol-5-ene
50
121
133
204
105
91
147
189
77
41
162
55
67
0
50
70
90
110
130
150
170
190
210
175
100
Peak 1
50
204
121
133
105
91
147
189
77
162
41
55
67
0
50
70
90
110
130
150
170
190
210
Ion abundance
Ion abundance
121
100
93
136
50
161
77
105
41
67
53
189
115
148
204
175
0
50
70
90
110
130
150
170
190
210
121
100
93
41
136
50
77
161
105
53
67
115
189
204
148
175
0
50
70
90
110
130
150
170
190
210
Peak 2
δ-eIemene
Ion abundance
Ion abundance
7-epi-Silphiperfol-5-ene
175
100
204
121
189
147
133
105
91
50
161
77
41
55
67
0
50
70
90
110
130
150
170
190
210
121
100
105
133
175
91
204
189
147
50
77
41
161
55
67
0
50
70
90
110
130
150
170
190
210
Peak 3
Ion abundance
Ion abundance
(+)-cyclosativene
161
100
105
119
204
94
133
50
41
189
79
147
55
67
175
0
50
70
90
110
130
150
170
190
210
105
100
161
119
91
204
94
50
41
79
133
189
55
67
147
175
0
50
70
90
110
130
150
170
190
210
Peak 4
Ion abundance
Ion abundance
161
100
119
105
50
93
204
81
41
133
55
189
69
147
175
0
50
70
90
110
130
150
170
190
210
161
100
119
105
50
91
41
204
77
133
55
69
189
147
175
0
50
70
90
110
130
150
170
190
210
Peak 5
α-copaene
Ion abundance
Ion abundance

## Slide 2
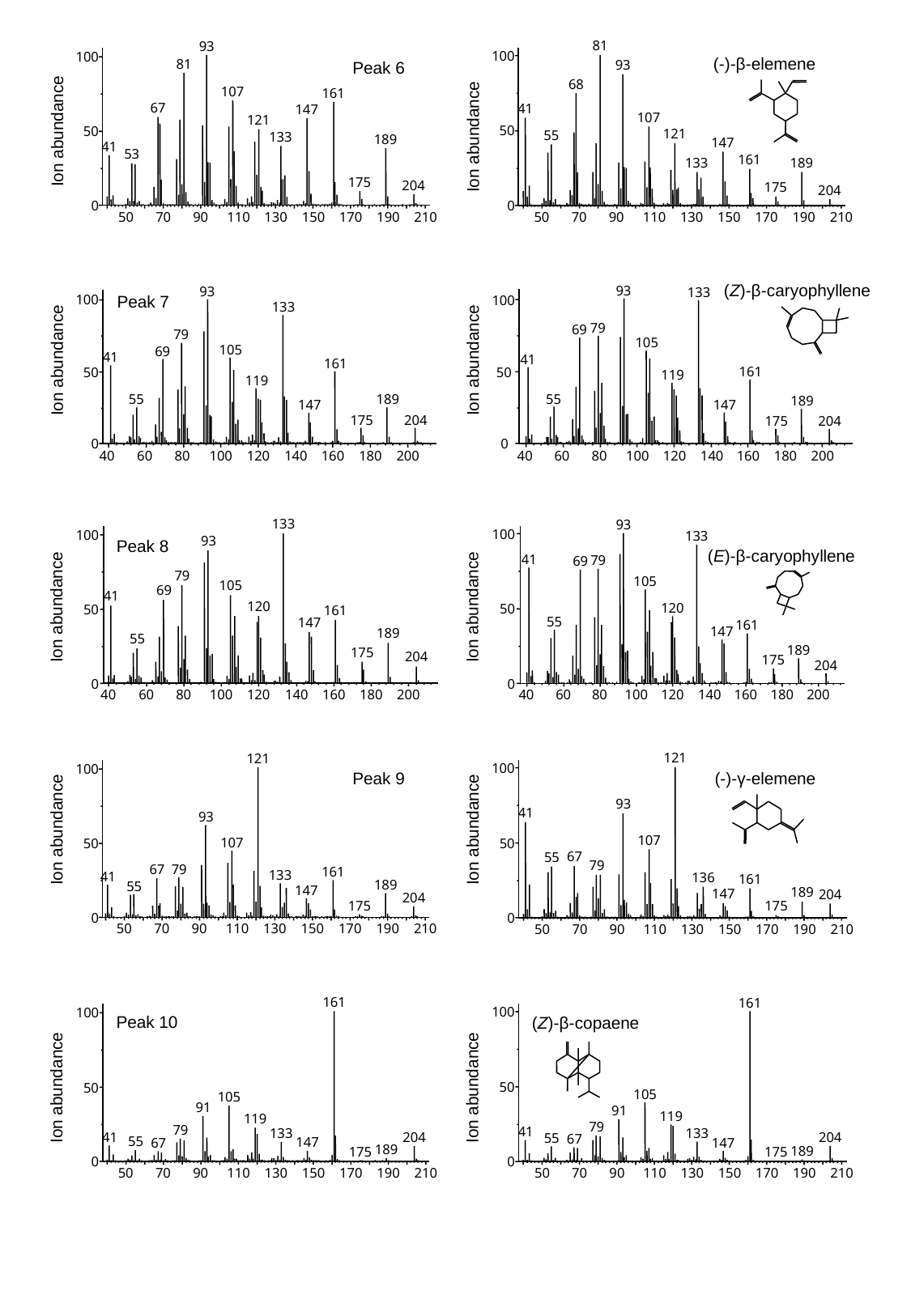

81
100
93
68
41
107
50
121
55
147
161
133
189
175
204
0
50
70
90
110
130
150
170
190
210
93
100
81
107
161
67
147
121
50
133
189
41
53
175
204
0
50
70
90
110
130
150
170
190
210
(-)-β-elemene
Peak 6
Ion abundance
Ion abundance
(Z)-β-caryophyllene
93
133
100
79
69
105
41
161
50
119
55
189
147
204
175
0
40
60
80
100
120
140
160
180
200
93
100
133
79
105
69
41
161
50
119
55
189
147
175
204
0
40
60
80
100
120
140
160
180
200
Peak 7
Ion abundance
Ion abundance
133
100
93
79
105
69
41
120
50
161
147
189
55
175
204
0
40
60
80
100
120
140
160
180
200
93
100
133
41
79
69
105
120
50
55
161
147
189
175
204
0
40
60
80
100
120
140
160
180
200
Peak 8
(E)-β-caryophyllene
Ion abundance
Ion abundance
121
100
93
41
107
50
67
55
79
136
161
189
147
204
175
0
50
70
90
110
130
150
170
190
210
121
100
93
107
50
79
67
161
133
41
189
55
147
204
175
0
50
70
90
110
130
150
170
190
210
Peak 9
(-)-γ-elemene
Ion abundance
Ion abundance
161
100
50
105
91
119
79
133
41
204
55
147
67
189
175
0
50
70
90
110
130
150
170
190
210
161
100
50
105
91
119
79
41
133
204
55
67
147
189
175
0
50
70
90
110
130
150
170
190
210
Peak 10
(Z)-β-copaene
Ion abundance
Ion abundance

## Slide 3
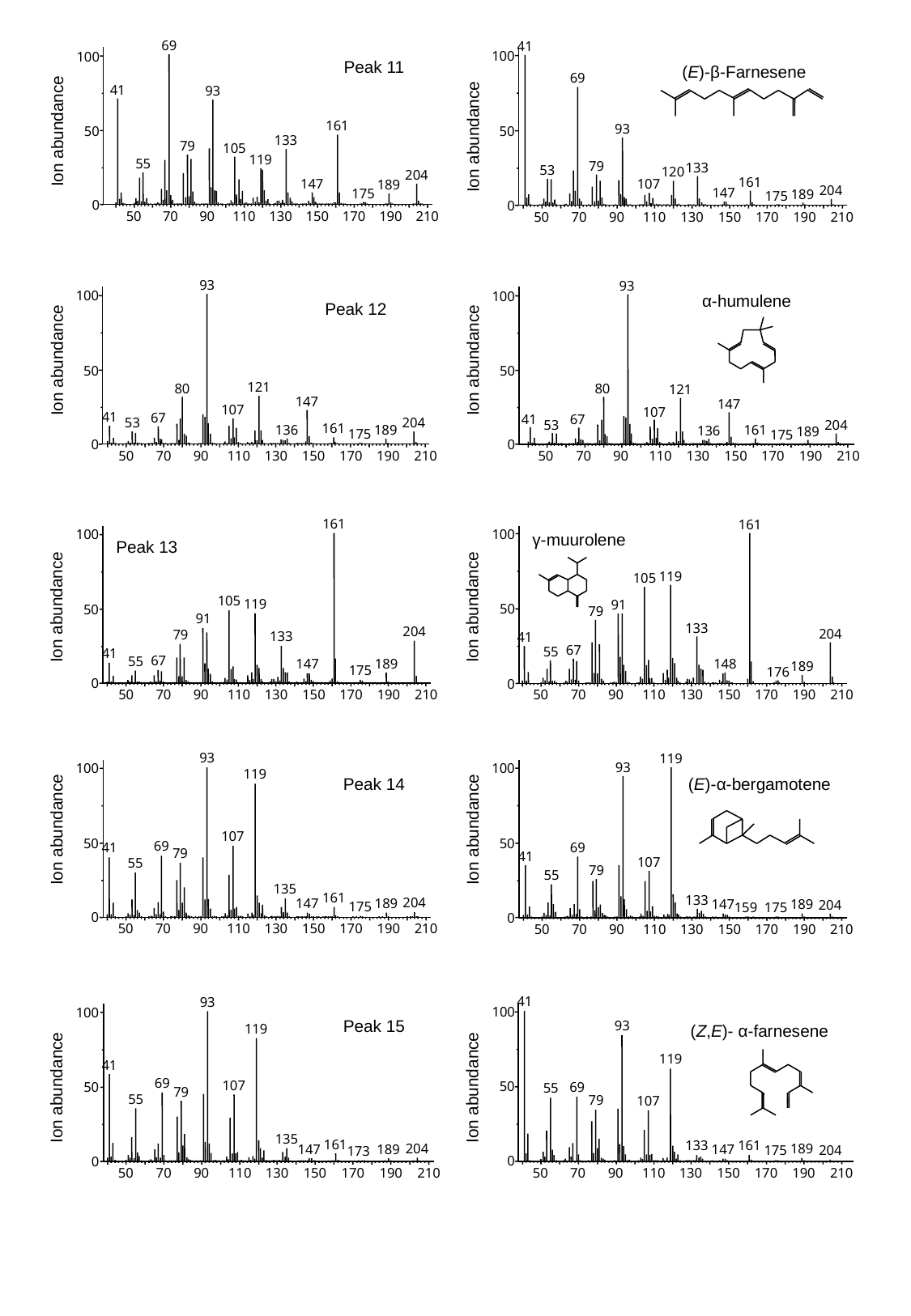

69
100
41
93
161
50
133
79
105
119
55
204
147
189
175
0
50
70
90
110
130
150
170
190
210
41
100
69
93
50
79
133
53
120
161
107
204
147
189
175
0
50
70
90
110
130
150
170
190
210
Peak 11
(E)-β-Farnesene
Ion abundance
Ion abundance
93
100
50
121
80
147
107
41
67
53
204
161
136
189
175
0
50
70
90
110
130
150
170
190
210
Peak 12
93
100
50
0
80
121
147
107
41
67
53
204
161
136
189
175
50
70
90
110
130
150
170
190
210
α-humulene
Ion abundance
Ion abundance
161
100
105
119
50
91
204
79
133
41
67
55
189
147
175
0
50
70
90
110
130
150
170
190
210
161
100
119
105
91
50
79
133
204
41
67
55
148
189
176
0
50
70
90
110
130
150
170
190
210
γ-muurolene
Peak 13
Ion abundance
Ion abundance
93
100
119
107
50
69
41
79
55
135
161
204
147
189
175
0
50
70
90
110
130
150
170
190
210
119
93
100
50
69
41
107
79
55
133
147
189
204
159
175
0
50
70
90
110
130
150
170
190
210
(E)-α-bergamotene
Peak 14
Ion abundance
Ion abundance
41
100
93
119
50
69
55
79
107
133
161
189
147
204
175
0
50
70
90
110
130
150
170
190
210
93
100
119
41
69
107
50
79
55
135
161
204
147
189
173
0
50
70
90
110
130
150
170
190
210
Peak 15
(Z,E)- α-farnesene
Ion abundance
Ion abundance

## Slide 4
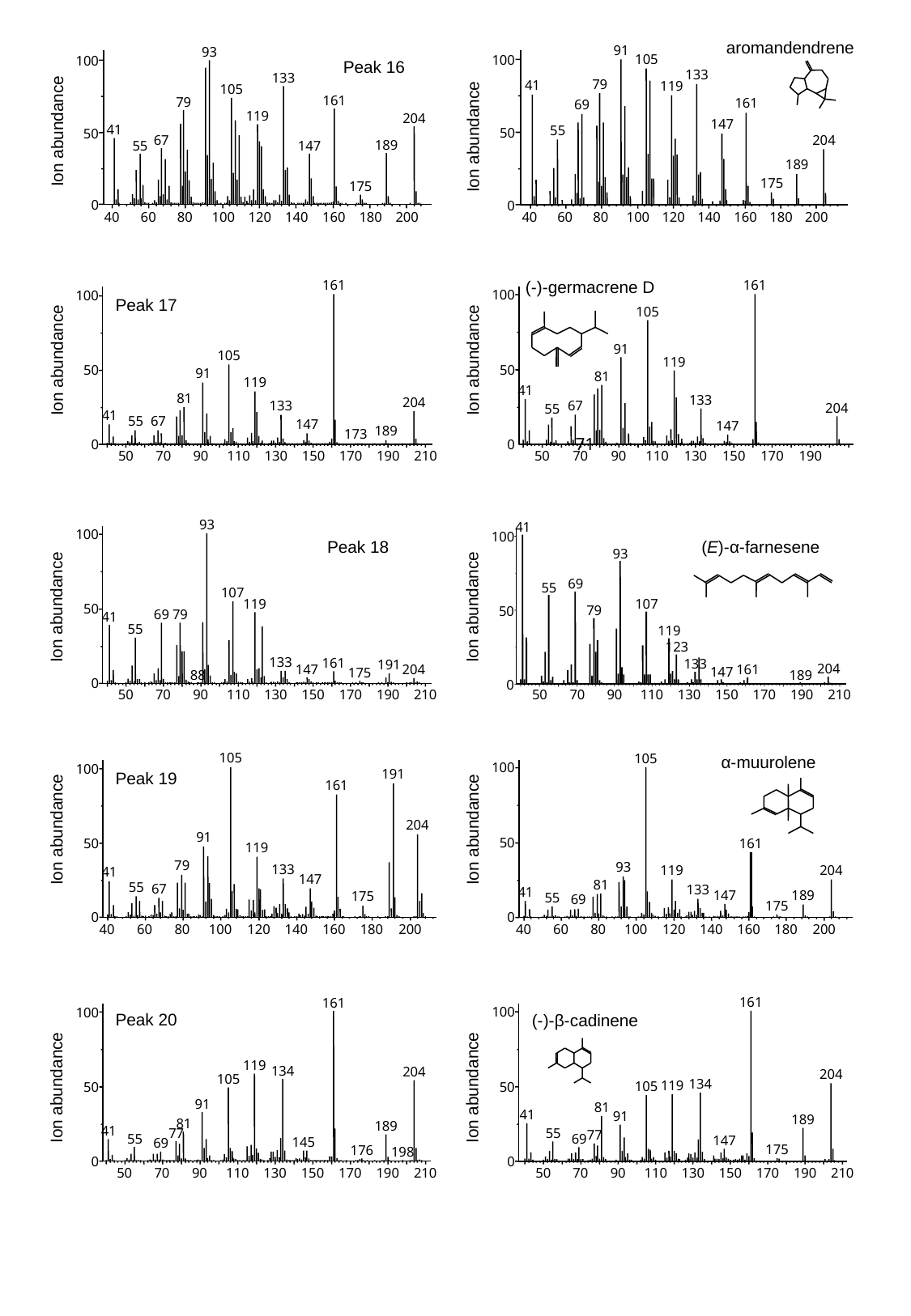

aromandendrene
91
105
100
133
79
41
119
161
69
147
55
50
204
189
175
0
40
60
80
100
120
140
160
180
200
93
100
133
105
161
79
119
204
41
50
67
189
55
147
175
0
60
80
100
120
140
160
180
200
40
Peak 16
Ion abundance
Ion abundance
(-)-germacrene D
161
100
105
50
91
119
81
204
133
41
55
67
147
189
173
0
50
70
90
110
130
150
170
190
210
161
100
105
91
119
50
81
41
133
67
204
55
147
71
0
50
70
90
110
130
150
170
190
Peak 17
Ion abundance
Ion abundance
93
100
107
119
50
69
79
41
55
133
161
191
147
204
175
88
0
50
70
90
110
130
150
170
190
210
41
93
69
55
107
79
50
119
123
133
204
161
147
189
0
50
70
90
110
130
150
170
190
210
100
Peak 18
(E)-α-farnesene
Ion abundance
Ion abundance
α-muurolene
105
100
191
161
204
91
50
119
79
133
41
147
55
67
175
0
40
60
80
100
120
140
160
180
200
105
100
50
161
93
119
204
81
133
41
147
189
55
69
175
0
40
60
80
100
120
140
160
180
200
Peak 19
Ion abundance
Ion abundance
161
100
204
134
119
105
50
81
41
91
189
55
77
69
147
175
0
50
70
90
110
130
150
170
190
210
161
100
119
134
204
105
50
91
81
189
41
77
55
145
69
176
198
0
50
70
90
110
130
150
170
190
210
Peak 20
(-)-β-cadinene
Ion abundance
Ion abundance

## Slide 5
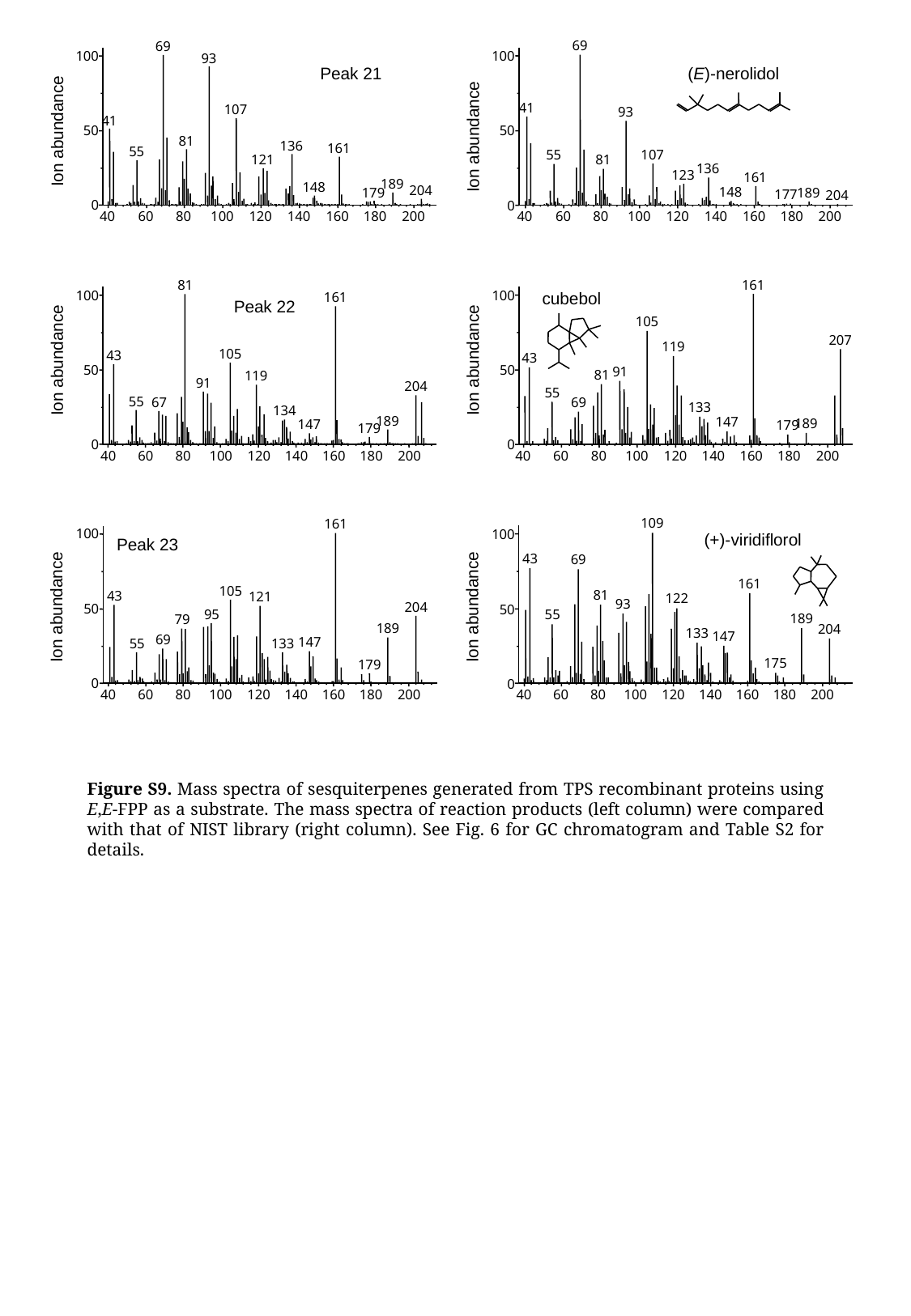

69
100
41
93
50
107
55
81
136
123
161
148
189
177
204
0
40
60
80
100
120
140
160
180
200
69
100
93
107
41
50
81
136
161
55
121
189
148
204
179
0
40
60
80
100
120
140
160
180
200
(E)-nerolidol
Peak 21
Ion abundance
Ion abundance
161
100
105
207
119
43
50
91
81
55
69
133
147
189
179
0
40
60
80
100
120
140
160
180
200
81
100
161
105
43
50
119
91
204
55
67
134
189
147
179
0
40
60
80
100
120
140
160
180
200
cubebol
Peak 22
Ion abundance
Ion abundance
109
100
43
69
161
81
122
93
50
55
189
204
133
147
175
0
40
60
80
100
120
140
160
180
200
161
100
105
43
121
204
50
95
79
189
69
147
55
133
179
0
40
60
80
100
120
140
160
180
200
(+)-viridiflorol
Peak 23
Ion abundance
Ion abundance
Figure S9. Mass spectra of sesquiterpenes generated from TPS recombinant proteins using E,E-FPP as a substrate. The mass spectra of reaction products (left column) were compared with that of NIST library (right column). See Fig. 6 for GC chromatogram and Table S2 for details.
