# Supplementary material for: Genomic Identification and Biochemical Characterization of Methyl Jasmonate (MJ)-Inducible Terpene Synthase Genes in Lettuce (Lactuca sativa L. cv. Salinas)
Source: Plants (Basel). 2025 Dec 24;15(1):55. doi: 10.3390/plants15010055 (PMC12787478; doi:10.3390/plants15010055)
Supplement: Supplementary file 1 [file plants-15-00055-s001.zip › Table S1. A list of LsTPS in lettuce.pdf]

Table S1. A list of *LsTPS* genes in *L. sativa* L. cv. Salinas

Designation[a]	Gene Model[b]	Chromosome			CDS[c]	AA[d]	Localization[e]	Sub family
		Chr	start	stop				
LsTPS1	*Lsat_1_v5_gn_0_28161*	Chr1	52259833	52269722	1572	524	Cytoplasm[T]	TPS-a
LsTPS2[p]	*Lsat_1_v5_gn_0_44201*	Chr6	83319999	83320462	381	127	Cytoplasm[T]	TPS-a
LsTPS3	*Lsat_1_v5_gn_0_8301*	Chr6	84318936	84322655	1470	490	Cytoplasm[T]	TPS-a
LsTPS4	*Lsat_1_v5_gn_1_115560*	Chr1	175263007	175270390	1683	561	Cytoplasm[T]	TPS-a
LsTPS5	*Lsat_1_v5_gn_1_121241*	Chr1	199170639	199175388	1683	561	Cytoplasm[T]	TPS-a
LsTPS6	*Lsat_1_v5_gn_1_123421*	Chr1	209785186	209788115	1683	561	Cytoplasm[T]	TPS-a
LsTPS7[p]	*Lsat_1_v5_gn_1_123481*	Chr1	198168665	198172751	489	163	Cytoplasm[T]	TPS-a
LsTPS8	*Lsat_1_v5_gn_1_123521*	Chr1	198142402	198145163	1683	560	Cytoplasm[T]	TPS-a
LsTPS9	*Lsat_1_v5_gn_1_127360*	Chr1	196368076	196375688	2532	844	Cytoplasm[T]	TPS-e/f
LsTPS10	*Lsat_1_v5_gn_1_5141*	Chr1	6154819	6158505	1644	548	Cytoplasm[T]	TPS-a
LsTPS11	*Lsat_1_v5_gn_1_5240*	Chr1	6077283	6082908	1572	524	Cytoplasm[T]	TPS-a
LsTPS12	*Lsat_1_v5_gn_1_84540*	Chr1	107964694	107967000	1566	522	Cytoplasm[T]	TPS-a
LsTPS13	*Lsat_1_v5_gn_2_110381*	Chr2	188502309	188511559	1785	595	Chloroplast[T]	TPS-b
LsTPS14	*Lsat_1_v5_gn_2_29221*	Chr2	65558523	65562622	1755	585	Cytoplasm[T]	TPS-a
LsTPS15	*Lsat_1_v5_gn_2_30320*	Chr2	67977247	67979916	1485	495	Cytoplasm[T]	TPS-b
LsTPS16	*Lsat_1_v5_gn_2_30961*	Chr2	67562066	67564808	1764	588	Mitochondria[T]	TPS-b
LsTPS17	*Lsat_1_v5_gn_3_23121*	Chr3	30351681	30354881	1674	558	Cytoplasm[T]	TPS-a
LsTPS18[p]	*Lsat_1_v5_gn_3_69740*	Chr3	90461013	90461417	309	103	Cytoplasm[T]	TPS-e/f
LsTPS19[p]	*Lsat_1_v5_gn_4_145200*	Chr4	285152904	285153342	306	102	Cytoplasm[T]	TPS-b
LsTPS20[p]	*Lsat_1_v5_gn_4_145220*	Chr4	285151535	285152864	576	192	Cytoplasm[T]	TPS-b
LsTPS21	*Lsat_1_v5_gn_4_145280*	Chr4	284905476	284907858	1782	593	Chloroplast[T]	TPS-b
LsTPS22	*Lsat_1_v5_gn_4_145300*	Chr4	284872363	284874388	1524	508	Cytoplasm[T]	TPS-b
LsTPS23	*Lsat_1_v5_gn_4_145381*	Chr4	285742929	285745325	1782	593	Chloroplast[T]	TPS-b
LsTPS24	*Lsat_1_v5_gn_4_170740*	Chr4	346257064	346259451	1548	516	Cytoplasm[T]	TPS-g
LsTPS25	*Lsat_1_v5_gn_4_30941*	Chr4	48010223	48014189	2457	819	Chloroplast[T]	TPS-e/f
LsTPS26	*Lsat_1_v5_gn_4_58020*	Chr4	87471610	87477282	1641	547	Cytoplasm[T]	TPS-b
LsTPS27	*Lsat_1_v5_gn_4_58141*	Chr4	87276479	87284798	1692	564	Cytoplasm[T]	TPS-g
LsTPS28	*Lsat_1_v5_gn_4_58221*	Chr4	87065301	87069576	1668	556	Cytoplasm[T]	TPS-g
LsTPS29	*Lsat_1_v5_gn_4_69040*	Chr4	106576850	106580215	1749	583	Chloroplast[T]	TPS-g
LsTPS30	*Lsat_1_v5_gn_5_40321*	Chr5	85173455	85176889	1179	393	Cytoplasm[T]	TPS-a
LsTPS31	*Lsat_1_v5_gn_5_42681*	Chr5	89407657	89410744	1707	569	Cytoplasm[T]	TPS-a
LsTPS32	*Lsat_1_v5_gn_6_21340*	Chr6	27646600	27649297	1518	506	Cytoplasm[T]	TPS-a
LsTPS33	*Lsat_1_v5_gn_6_23080*	Chr6	30181268	30183875	1617	539	Cytoplasm[T]	TPS-a
LsTPS34	*Lsat_1_v5_gn_6_32141*	Chr6	41807206	41818156	1659	553	Cytoplasm[T]	TPS-b
LsTPS35	*Lsat_1_v5_gn_6_32320*	Chr6	41927736	41931903	1545	515	Cytoplasm[T]	TPS-b
LsTPS36	*Lsat_1_v5_gn_6_33321*	Chr6	42240666	42248591	1884	628	Cytoplasm[T]	TPS-b
LsTPS37[p]	*Lsat_1_v5_gn_6_33341*	Chr6	42130520	42130804	285	95	Cytoplasm[T]	TPS-b
LsTPS38[p]	*Lsat_1_v5_gn_6_33381*	Chr6	42091602	42091928	327	109	Cytoplasm[T]	TPS-b
LsTPS39	*Lsat_1_v5_gn_6_58961*	Chr6	83076376	83081195	1653	551	Cytoplasm[T]	TPS-a
LsTPS40[p]	*Lsat_1_v5_gn_6_58980*	Chr6	83300669	83301742	366	122	Cytoplasm[T]	TPS-a
LsTPS41	*Lsat_1_v5_gn_6_59741*	Chr6	83321258	83330547	1731	577	Cytoplasm[T]	TPS-a
LsTPS42	*Lsat_1_v5_gn_8_116340*	Chr8	178016363	178019730	1674	558	Cytoplasm[T]	TPS-a
LsTPS43	*Lsat_1_v5_gn_8_116421*	Chr8	178129666	178132947	1680	560	Cytoplasm[T]	TPS-a
LsTPS44	*Lsat_1_v5_gn_8_142801*	Chr8	237165238	237167753	1641	547	Cytoplasm[T]	TPS-b
LsTPS45[p]	*Lsat_1_v5_gn_8_142820*	Chr8	236824427	236824746	237	79	Cytoplasm[T]	TPS-b
LsTPS46[p]	*Lsat_1_v5_gn_8_142841*	Chr8	236821590	236822620	729	243	Cytoplasm[T]	TPS-b
LsTPS47	*Lsat_1_v5_gn_8_142860*	Chr8	236815076	236817558	1758	586	Mitochondria[T]	TPS-b
LsTPS48	*Lsat_1_v5_gn_8_143440*	Chr8	237893233	237895693	1518	506	Cytoplasm[T]	TPS-b
LsTPS49[p]	*Lsat_1_v5_gn_8_143560*	Chr8	237758226	237761187	504	168	Cytoplasm[T]	TPS-b
LsTPS50	*Lsat_1_v5_gn_8_143701*	Chr8	238443459	238446089	1758	586	Mitochondria[T]	TPS-b
LsTPS51	*Lsat_1_v5_gn_9_20260*	Chr9	21299944	21307332	1602	534	Cytoplasm[T]	TPS-a
LsTPS52	*Lsat_1_v5_gn_9_20320*	Chr9	21373718	21376162	1650	550	Cytoplasm[T]	TPS-a
LsTPS53	*Lsat_1_v5_gn_9_20380*	Chr9	21418269	21421018	1533	511	Cytoplasm[T]	TPS-a
LsTPS54	*Lsat_1_v5_gn_9_3541*	Chr9	1922421	1928072	2364	788	Chloroplast[T]	TPS-e/f

a - Designation is annotated according to gene ID; b - Gene I.D. is annotated from phytozome database; c - Coding sequence in nucleotides; d - Amino acids; e - Subcellular localization predicted by [T]TargetP2.0 (Emanuelsson et al., 2007); p - Pseudogene

- *LsTPS14*, *LsTPS42*, *LsTPS43* - *germacrene A synthase 3, 2, 1*, respectively (Kwon et al., 2022; Bennett et al., 2002)

- *LsTPS25*, *LsTPS54* - *ent-kaurene synthase* and *ent-copalyl diphosphate synthase 1*, respectively (Sawada et al., 2008)
