# Supplementary material for: Genomic Identification and Biochemical Characterization of Methyl Jasmonate (MJ)-Inducible Terpene Synthase Genes in Lettuce (Lactuca sativa L. cv. Salinas)
Source: Plants (Basel). 2025 Dec 24;15(1):55. doi: 10.3390/plants15010055 (PMC12787478; doi:10.3390/plants15010055)
Supplement: Supplementary file 1 [file plants-15-00055-s001.zip › Table S3. RT-PCR primers.pdf]

Supplementary Table S3. Primer sequences used for RT-PCR. Gene I.D. represent respective terpene synthase genes annotated in table 1. *L. sativa Tubulin*, *LOC111880334*; and *L. sativa Actin*, *LOC111882438*; were used as endogenous control for qualitative analysis.

Gene I.D.	Primer sequences (forward / reverse)
LsTPS1	5'- AGGCTTCTTCGTCTCAAGTGAT -3'/<br>5'- CAGCAGAAACAACAGGGTCG -3'
LsTPS3	5'- TGATGGAATGTAGCAAGCCG -3'/<br>5'- TGTCGCATGAGTCGAAACCA -3'
LsTPS4	5'- GTGAGACAGGCGTTGAGGAG -3'/<br>5'- CCCAACTGTTTATGGCACCC -3'
LsTPS5	5'- TGACTATTTGGAGTTGGAAGCA -3'/<br>5'- GAGCTTCTGTGAAGACGATAGC -3'
LsTPS6	5'- TGAGGGTGAAAGAGCACAGG -3'/<br>5'- AAGAACCGGTGCAACTAGCC -3'
LsTPS8	5'- TGAAGAATGGCGAAGAGGCT -3'/<br>5'- GCCACGTTTTCCGTTAACCC -3'
LsTPS9	5'- AACGTTTCTCGAGGGGAAGC -3'/<br>5'- TCATGGACTTCGTGGGAAGC -3'
LsTPS10	5'- GACTTGAGGCTTTGCGCTAC -3'/<br>5'- CTTGCATCGCCTCTTGAACA -3'
LsTPS11	5'- TCCAACAACCGAAGAGCACA -3'/<br>5'- GGTTTTGCACTTCTTCCCCG -3'
LsTPS12	5'- ACGAACGCCAGACCTTTCAT -3'/<br>5'- TATTCCGGCAGGTCTTCCAC -3'
LsTPS13	5'- TAAGCCATGCCTTGGAGCTT -3'/<br>5'- CTGGTTGATCAGGGTGCTGT -3'
LsTPS14	5'- ATCGGCTACGGGTGTTGATG -3'/<br>5'- CCATAAAACATCCCAAAAGAGACCA -3'
LsTPS15	5'- TGAGCTCGCCAAATTGGACT -3'/<br>5'- GATCACGAGCAAAGCCGAAC -3'
LsTPS16	5'- AGAGTGGTGCCACAGAAGTG -3'/<br>5'- AACAAAATACCCTAATGCTCACAA -3'
LsTPS17	5'- GATCGAGAATGCGAGGAGCA -3'/<br>5'- ATGAAACTCCCGTCGCTGTT -3'
LsTPS21	5'- CAAGGATGGTCATGGTGGCT -3'/<br>5'- TTGCAAGCTCCAACACATGC -3'
LsTPS22	5'- AGTGTGGGGAGATTTACTTGAAGC -3'/<br>5'- TCTCCGTATTGGTACACGCAT -3'
LsTPS23	5'- GCTGTCAAGAGGTGGGACAT -3'/<br>5'- GCAACTTTCTCACACGCACC -3'
LsTPS24	5'- TGTTGAGCAGGAAGACAACGA -3'/<br>5'- TCCTCTCCTGCAAAATCTGGT -3'
LsTPS25	5'- CGACTTGAACTCAAGGGGACA -3'/<br>5'- GGCTACCCAAGCTGTGTCAT -3'
LsTPS26	5'- TGGTTGGGTGACATCATGTGG -3'/

	5'- TAGTCTCACGATCTTGGATGCC -3'
LsTPS27	5'- TCAAGTTGTCGGGCGGTAAA -3'/<br>5'- GCACACCTTCGACATGCTTT -3'
LsTPS28	5'- CCAGGCCCTACATCGAACAG -3'/<br>5'- AGCCTCAATACATATCCTGAAGTG -3'
LsTPS29	5'- CGAGATCGGCCTCTTGAGTG -3'/<br>5'- GCCATGTATGTCCCTGCTGT -3'
LsTPS30	5'- GACGTTCACAGAGAAGCCGA -3'/<br>5'- AAGCTGTCACCAGAGGAGGA -3'
LsTPS31	5'- AGCTTGGGAGGAGCAACAAG -3'/<br>5'- ACGGAAGTGCTTTGGAGGTT -3'
LsTPS32	5'- TATTGTCACCCACAAGGGCG -3'/<br>5'- ACATCCATTCCTCGTCCCAA -3'
LsTPS33	5'- GCTAAGGCTATGGAGGAGCC -3'/<br>5'- TGCGTGTTTCACTTGCTGTG -3'
LsTPS34	5'- TGCCACAGAAGTGGAAGCAA -3'/<br>5'- TCATACGCAAAGTGGCCCAT -3'
LsTPS35	5'- CGAGGATGTGGTGGGAATGT -3'/<br>5'- TTCCACTAAGCGGTCCCGAG -3'
LsTPS36	5'- GAAGGGGCTCAACTCTACTCG -3'/<br>5'- AGTCTCCCAAGCAATGCCTC -3'
LsTPS39	5'- GAGAGCTCGATCGGTGAGTG -3'/<br>5'- TGTCGCATGAGTCGAAACCA -3'
LsTPS41	5'- TCTTTTGTTGGCATGGGGGA -3'/<br>5'- TTTGAACCGTGTGGCGTAGT -3'
LsTPS42	5'- TCAACCAATCACCATTCCACA -3'/<br>5'- GGCTTCCATGGCTTTTGCAT -3'
LsTPS43	5'- AAGGGGTCAATCAGCCACTG -3'/<br>5'- TGACATAAATTGATATACCCGACCA -3'
LsTPS44	5'- TGCCAGAGACAGATTGGTGG -3'/<br>5'- TACTGAAGCGCCTATGGATAC -3'
LsTPS47	5'- AGATTCTTATGCCAAAAGCGGAG -3'/<br>5'- ATTTCGAGTTGTGTCGGTGA -3'
LsTPS48	5'- TCGGTAGGGGCTCCTGTAAT -3'/<br>5'- GCACCTCCTCGGGCTTTATT -3'
LsTPS50	5'- CCTGTGGACAGTTGGGGTG -3'/<br>5'- GTAGCGAACTATATTTTCAGCTCTC -3'
LsTPS51	5'- CTCCAAGCGTTTGGGGAGAT -3'/<br>5'- TCCTTTGCTATTTCTTCAAGGTGA -3'
LsTPS52	5'- AGACTTGAAACAAGAAGTGAGGG -3'/<br>5'- AGGCTGCTTTAGTGCCTCTT -3'
LsTPS53	5'- TTGGGCAGTCTGTGTATGCC -3'/<br>5'- TCGCAATATCACCCATGCCG -3'
LsTPS54	5'- CTTCGGTGCAAAAGCTGTGT -3'/<br>5'- AAGGACTACACTGCCTGCC -3'
LsHMGR	5'- GGTCGTGGGAAATCTGTGGT -3'/<br>5'- AGCATGCGCATTGAAACCTC -3'
LsDXR	5'- CAGGACTCGTCGGTTCTAGC -3'/

	5'- CGGCCGAATAAGCCAAATCC -3'
LsTUB	5'- TAGGCGTGTGAGTGAGCAGT -3'/<br>5'- AACCCTCGTACTCTGCTGCCTCTT -3'
LsACT	5'- AGGGCAGTGTTTCCTAGTATTGTT -3'/<br>5'- CTCTTTTGGATTGTGCCTCATCT -3'
