# Supplementary material for: Genomic Identification and Biochemical Characterization of Methyl Jasmonate (MJ)-Inducible Terpene Synthase Genes in Lettuce (Lactuca sativa L. cv. Salinas)
Source: Plants (Basel). 2025 Dec 24;15(1):55. doi: 10.3390/plants15010055 (PMC12787478; doi:10.3390/plants15010055)
Supplement: Supplementary file 1 [file plants-15-00055-s001.zip › Table S4. qRT-PCR primers.pdf]

Supplementary Table S4. Primer sequences used for qRT-PCR. Gene I.D. represent respective terpene synthase genes annotated in table 1. *L. sativa Tubulin*, *LOC111880334*; was used as endogenous control for quantitative analysis.

Gene I.D.	Primer sequences (forward / reverse)
LsTPS4	5'- AGGCTTCTTCGTCTCAAGTGAT -3'/<br>5'- CAGCAGAAACAACAGGGTCG -3'
LsTPS5	5'- TAGCACAGATCCAGTGCGTC -3'/<br>5'- AGAGAGTTGGAAGCATACGC -3'
LsTPS8	5'- TGAAGAATGGCGAAGAGGCT -3'/<br>5'- GCCACGTTTTCCGTTAACCC -3'
LsTPS10	5'- TGCCATGGGTGATGCGATAA -3'/<br>5'- GCTCTTCCTTGTGGGTGACA -3'
LsTPS15	5'- CTTCACAGCAACGCAAAGCA -3'/<br>5'- ATACTTCCCACCCAAGCGAT -3'
LsTPS16	5'- AATCCAATCCAAGGGTTAGCA -3'/<br>5'- AACAAAATACCCTAATGCTCACAA -3'
LsTPS21	5'- CAAGGATGGTCATGGTGGCT -3'/<br>5'- TGGCCTCGTGCAAATCTCTT -3'
LsTPS23	5'- GAGAGTGGTGCGTGTGAGAA -3'/<br>5'- TTGAGAACAAGCCACTCGGG -3'
LsTPS24	5'- AAACGTTGCCGTTTTGCAGT -3'/<br>5'- CCAGGTCCTTTGACCACCTT -3'
LsTPS26	5'- AAGGGACATCAAAGGCGGAG -3'/<br>5'- AAAATGTGCAACTCGAGCCA -3'
LsTPS28	5'- ACTCACACCAGAGTAGGTGA -3'/<br>5'- GTCTAGTACCCGATACGCACT -3'
LsTPS34	5'- ATGGGCCACTTTGCGTATGA -3'/<br>5'- GTTACGGCACATGCAAGACA -3'
LsTPS42	5'- CAAAAGCCATGGAAGCCCCAAA -3'/<br>5'- AAACCAAGACGGTGGACGGAATA -3'
LsTPS43	5'- ATCCACACAACACCTCAAGTT -3'/<br>5'- GGGAAATTGGCCAAAGGACG -3'
LsTPS50	5'- GATGACTTAGGGACATCCTCGG -3'/<br>5'- AAAACTGTGAGTTTGCGCCT -3'
LsTPS51	5'- TTCACCTTGAAGAAATAGCAAAGG -3'/<br>5'- TGTAGCGCAAAGCCTCTAGT -3'
LsTPS52	5'- AGACTTGAAACAAGAAGTGAGGG -3'/<br>5'- CCCCATTCCAGTCATCACCAT -3'
LsHMGR	5'- GGTCGTGGGAAATCTGTGGT -3'/<br>5'- AGCATGCGCATTGAAACCTC -3'
LsDXR	5'- CAGGACTCGTCGGTTCTAGC -3'/<br>5'- CGGCCGAATAAGCCAAATCC -3'
LsTub	5'- TAGGCGTGTGAGTGAGCAGT -3'/<br>5'- AACCCTCGTACTCTGCTGCCTCTT -3'
