# Supplementary material for: Genomic Identification and Biochemical Characterization of Methyl Jasmonate (MJ)-Inducible Terpene Synthase Genes in Lettuce (Lactuca sativa L. cv. Salinas)
Source: Plants (Basel). 2025 Dec 24;15(1):55. doi: 10.3390/plants15010055 (PMC12787478; doi:10.3390/plants15010055)
Supplement: Supplementary file 1 [file plants-15-00055-s001.zip › Table S5. Primers for expression vector cloning.pdf]

Supplementary Table S5. Primer sequences used for cloning of *LsTPS* genes for recombinant protein expression in bacterial system. Gene I.D. represent respective terpene synthase genes annotated in table 1 and restriction enzymes are underlined for each primer.

Gene I.D.	Primer sequences (forward / reverse)	Restriction enzyme
LsTPS8	5'- *GCTAGC*ATGCGTCCTTATGTCAATTTTCC-3'/<br>5'- *CTCGAG*TCAGTACATGGGAACAGAAGTTATG-3'	*NheI*<br>*XhoI*
LsTPS10	5'- *GCTAGC*ATGCGACCTACTGCTAATTTTC-3'/<br>5'- *CTCGAG*TCATGTGATGATAGCGTCAAC-3'	*NheI*<br>*XhoI*
LsTPS16	5'- *GGATCC*ATGAGAAGATCAGCAAATTA -3'/<br>5'- GCC*GTCGAC*TTATGCTAACCCTTGG -3'	*BamHI*<br>*SalI*
LsTPS21	5'- *GCTAGC*ATGAACTCCAGAGAGGAG -3'/<br>5'- *GGATCC*CTAATTATCCTTAATTGGAATGGG -3'	*NheI*<br>*BamHI*
LsTPS23	5'- *GGATCC*ATGGCGGACAAGAAC-3'/<br>5'- *GTCGAC*CTAATTATCCTTAATT-3'	*BamHI*<br>*SalI*
LsTPS24	5'- *GCTAGC*ATGAACAGTATTATTG -3'/<br>5'- *GGATCC*TCACATCGTACTATTC -3'	*NheI*<br>*BamHI*
